# pHusion – a robust and versatile toolset for automated detection and analysis of exocytosis

**DOI:** 10.1242/jcs.261828

**Published:** 2024-06-07

**Authors:** Ellen C. O'Shaughnessy, Mable Lam, Samantha E. Ryken, Theresa Wiesner, Kimberly Lukasik, J. Bradley Zuchero, Christophe Leterrier, David Adalsteinsson, Stephanie L. Gupton

**Affiliations:** ^1^ University of North Carolina at Chapel Hill, Department of Cell Biology and Physiology, Chapel Hill, NC 27599, USA; ^2^ Department of Neurosurgery, Stanford University School of Medicine, Stanford, CA 94305, USA; ^3^ NeuroCyto, Aix Marseille Université, CNRS, INP UMR7051, Marseille 13385, France; ^4^ University of North Carolina at Chapel Hill, Department of Mathematics, Chapel Hill, NC 27599, USA

**Keywords:** Exocytosis, Image analysis, Microscopy, Spatiotemporal analysis

## Abstract

Exocytosis is a fundamental process used by eukaryotes to regulate the composition of the plasma membrane and facilitate cell–cell communication. To investigate exocytosis in neuronal morphogenesis, previously we developed computational tools with a graphical user interface to enable the automatic detection and analysis of exocytic events from fluorescence timelapse images. Although these tools were useful, we found the code was brittle and not easily adapted to different experimental conditions. Here, we developed and validated a robust and versatile toolkit, named pHusion, for the analysis of exocytosis, written in ImageTank, a graphical programming language that combines image visualization and numerical methods. We tested pHusion using a variety of imaging modalities and pH-sensitive fluorophores, diverse cell types and various exocytic markers, to generate a flexible and intuitive package. Using this system, we show that VAMP3-mediated exocytosis occurs 30-times more frequently in melanoma cells compared with primary oligodendrocytes, that VAMP2-mediated fusion events in mature rat hippocampal neurons are longer lasting than those in immature murine cortical neurons, and that exocytic events are clustered in space yet random in time in developing cortical neurons.

## Teaser

**Summary:** A flexible image analysis tool for detection and spatiotemporal analysis of exocytosis that works across imaging modalities, pH-sensitive fluorophores, and diverse vesicle and cell types.

## INTRODUCTION

Exocytosis is an essential biological process during which secretory vesicles fuse with the plasma membrane (PM) thereby altering the composition of the PM and releasing lumenal cargo into the extracellular space. Although exocytosis is a common behavior in all eukaryotic cells, it serves myriad functions from altering membrane fluidity and changing cell shape to facilitating synaptic transmission, hormone release and remodeling the extracellular matrix (Burgoyne and Morgan, 2003; Lin and Scheller, 2000; Mostov et al., 2000; Winkle et al., 2014). The soluble N-ethylmaleimide-sensitive factor attachment proteins receptors (SNARE) complex is the minimal protein machinery required for exocytosis (Block et al., 1988; Jahn and Fasshauer, 2012; Südhof and Rothman, 2009; Weber et al., 1998). To enable fusion of membranes, SNARE proteins attached to different membranes form a tightly packed bundle of four α-helical coiled-coils (CCs) (Gao et al., 2012; Söllner et al., 1993; Sutton et al., 1998). Vesicle SNAREs (vSNAREs), such as the vesicle associated membrane protein (VAMP) family, contribute one α-helix and plasma membrane-targeted SNAREs (tSNARES), such as the syntaxin1 and SNAP25 families of proteins, contribute the other three α-helices (Söllner et al., 1993). Which SNAREs are bundled in a SNARE complex is a tightly regulated process that depends heavily on the cell type, developmental stage and exocytic mode (Urbina and Gupton, 2020). In neurons, two distinct modes of exocytosis have been elucidated: full vesicle fusion (FVF), in which membrane material is delivered to the plasma membrane, and kiss-and-run (KNR), in which vesicular cargo is released into the extracellular environment with no membrane incorporation (Bowser and Khakh, 2007; Harata et al., 2001; Heuser and Reese, 1973; Urbina et al., 2021). Although this core machinery involved in vesicle fusion has been elucidated over the past 40 years, understanding how these diverse processes are regulated remains an active area of investigation.

Recent advances in microscopy and better fluorescent tools to visualize exocytosis have given rise to large, image-based datasets that are poised to address previously inaccessible questions. Despite the availability of high-quality imaging data, image analysis frequently presents a bottleneck to discovery. Manual image analysis is problematic as it is error prone, subject to bias and time consuming. To overcome these limitations in our work studying neuronal morphogenesis, we previously developed ADAE GUI, a semi-automated computer-vision based toolkit using MATLAB (MathWorks Inc.; https://www.mathworks.com) and The R Project for Statistical Computing (https://www.r-project.org/) to identify exocytic events, characterize their spatio-temporal dynamics and classify distinct modes of exocytosis (Urbina and Gupton, 2021; Urbina et al., 2018, 2021).

Although these computational tools were instrumental in several studies (Lam et al., 2022; Urbina et al., 2018, 2021; Ye et al., 2022), we have found that different modes of microscopy, such as total internal reflection fluorescence (TIRF) versus widefield epifluorescence microscopy, different camera sensor technologies, tagging different vSNARE proteins with the commonly used fluorophore pHluorin or use of alternate pH-sensitive fluorescent proteins greatly impacted the functionality of this code. Unfortunately, the intermediate steps in ADAE GUI are opaque, both in terms of the code and the inability to visualize image transformations, making it challenging to identify and address points of failure. In addition, collaborators studying disparate cell types and/or distinct stages of neuronal development have struggled to implement the code and adapt it to their unique studies. Furthermore, ADAE GUI was unable to process movies over 4.29GB which, given larger camera chips and faster imaging capacity, have become common and therefore must be addressed. Finally, batch processing of datasets proved problematic, impacting usability.

To build a more robust and versatile toolset for automated detection and unbiased analysis of exocytosis, we have developed a workflow in ImageTank (https://www.visualdatatools.com/ImageTank/; O'Shaughnessy et al., 2019), that we have called pHusion. The requirements for use of pHusion are an ImageTank license, Xcode, GitHub and a computer running MacOS. ImageTank is a graphical programming language that combines image visualization with efficient numerical methods and an integrated interface to implement external code written in C^++^ (Stroustrup, 2013) or Python (https://www.python.org/). An advantage of this approach is that data are not transferred manually between separate applications as in our previous method, thereby reducing the risk of error and simplifying the process for the user. Further, pHusion is a script that can be saved for each cell (or group of cells) so that the input images, settings used and output information are contained in one place. The data flow architecture employed by ImageTank enables visual inspection of each data transformation or calculation in real time, facilitating the identification of appropriate parameters and detection of points of failure. Furthermore, to distinguish valid exocytic events, our previous analysis tools relied on particle-tracking algorithms that can be opaque and unintuitive to adapt for different datasets. Instead of using these tools, we defined a set of relatively simple criteria, such as permissible *x-y* drift and event duration, that can be easily visualized and adjusted based on the experimental design.

Finally, as in previous work, we employ Ripley's K-based analysis (Ripley, 1976, 1977; Urbina et al., 2018) to determine whether the spatial distribution of vesicle fusion events are clustered, uniform or dispersed. Previously, our automated analysis made assumptions regarding the shape of the cellular or subcellular region to account for edge effects in the analysis (Urbina et al., 2018). Here, we present a fully generalizable approach in which no assumptions regarding cellular shape are made. Instead, we rely entirely on experimentally determined cell segmentations. This more general method is better suited to the disparate cell types presented in this work and is thus more versatile. Furthermore, here we have implemented temporal analysis based on Poisson processes to assess whether exocytosis occurs in random bursts or is regulated in time.

Given the importance of vesicle fusion and the availability of high quality image-based datasets, a number of tools have recently been published to analyze exocytosis in an automated (Mahmood et al., 2023; Schmied et al., 2021) or semi-automated fashion (Bebelman et al., 2020). These powerful applications highlight the need for and interest in unbiased, computer-aided approaches to studying vesicle fusion that are also sufficiently robust for analysis of the diverse datasets. Our goal in the work presented here was to incorporate both the analysis we have come to rely on in our previous software to identify exocytic events and the characterization of spatio-temporal dynamics in a single application capable of handling diverse datasets, obviating the need to pass data between software and improving visualization capabilities. To develop this more robust and flexible processing pipeline, we have characterized exocytosis in diverse cell types, including developing primary murine cortical neurons at 2 days *in vitro* (DIV), mature rat hippocampal neurons (DIV12–14), a human melanoma cell line (1205^Lu^) and primary rat oligodendrocytes. Further, we have used different pH-sensitive fluorophores (sepHluorin and pHmScarlet) tagged to various vSNAREs (VAMP2, VAMP3 and VAMP7) imaged with TIRF microscopy, highly inclined and laminated optical sheet (HILO) microscopy, and widefield microscopy, and different camera sensor technologies. pHusion was adjusted for individual datasets and worked reliably in each scenario. We were able to capture markedly different frequencies of VAMP3-mediated exocytosis between oligodendrocytes and melanoma cells. Additionally, we demonstrate that the duration of constitutive VAMP2-mediated exocytic events in mature rat hippocampal neurons is much longer than that of immature murine cortical neurons.

## RESULTS

**Fig. 1.**
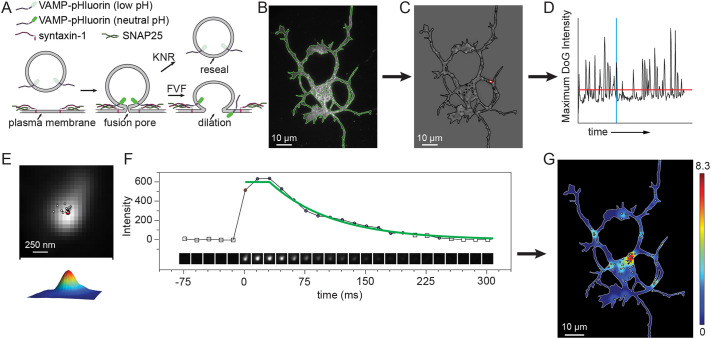
**Schematic of pHusion workflow.** (A) Schematic of pH-sensitive sepHluorin fused to a vSNARE, with full vesicle fusion (FVF) and kiss-and-run (KNR) modes of fusion depicted. (B) The images were segmented (green line) on the first frame and preprocessed as described in the text. (C) Example difference of Gaussian (DoG) image with region of interest highlighted in red. (D) The maximum intensity in the DoG image plotted over time. The threshold, shown as a red horizontal line is used to identify regions to evaluate further. The red contour shown in C is the region above the threshold for that time point. The vertical blue line identifies the time point displayed. (E) A Gaussian model was fitted to each cropped image and the center of the peak was used to quantify drift. Only those images with a goodness of fit above a defined value are included in the calculation, these timepoints are shown as circles in F. (F) Example of an image series centered on a region of interest, the mean intensity in each image and the fit to the intensity values (green line) from the start of the event. (G) Summary of all events detected and their spatial distribution.

To image exocytosis, we employed a pH-sensitive variant of GFP, superecliptic-pHluorin (sepHluorin) fused to the vSNARE VAMP2. sepHluorin is quenched in the acidic lumen of the vesicle and fluoresces rapidly following fusion and exposure to the neutral pH of the extracellular environment (Fig. 1A). After fusion, fluorescence decays exponentially as VAMP2–sepHluorin either diffuses in the plasma membrane or is quenched following vesicle closure and reacidification (Bowser and Khakh, 2007; Urbina et al., 2021). Imaging VAMP2–sepHluorin in developing cortical neurons, we found that our initial analysis pipeline, ADAE GUI, performed within ImageJ, MATLAB and R software was brittle, failing frequently, often for very minor changes in experimental data. To build a more robust and transparent analysis package, we used ImageTank for generalized image processing functions and custom code written in C^++^ for more specialized actions specific to exocytosis. The external code is launched from within ImageTank and results are passed back to it for further processing steps, thus avoiding the need to manually transfer data between separate applications. All computational steps, including spatio-temporal analysis, are performed in a single script using only the raw images in either the .tiff or .dtbin file format as input. For our initial work to develop pHusion, we used immature primary cortical murine neurons expressing VAMP2–sepHluorin as a model system.

### pHusion workflow

We started with the basic framework for identifying potential exocytic events established in our previous work (Urbina et al., 2018) and perform all computational steps in pHusion. Briefly, the cell was segmented on the first frame, as the movies were short enough to ignore minor changes in shape (Fig. 1B). The raw data were preprocessed to subtract the background and then photobleach corrected by histogram matching to the fluorescence intensity of the first frame. Bright stationary objects were removed by subtracting a rolling window median projection of the preceding five frames. To filter out noise and enhance bright regions with the Gaussian profile of meaningful fluorescence, we used a difference of Gaussian (DoG) approach, subtracting successively blurred images and taking the median projection of the resulting images (Fig. 1C). The initial level of blur (sigma) is specified by the user in pixels. The maximum intensity in each projection was plotted over time and a threshold was applied to identify regions to investigate further (Fig. 1D, red line). Alhough we followed the same basic steps as our previous work, a crucial difference here is that we perform all calculations on the data as floating-point numbers, allowing decimals, not integer values. We chose to use floating-point numbers as maintaining only integers reduced the complexity of the data too much to enable fine-tuning the threshold parameter and was a frequent source of inaccurate identification (Fig. S1). Furthermore, in the ADAE GUI images were reduced to 8-bit images to increase processing speed and reduce data size. Although useful, this further loss of data was unnecessary given the improved efficiency of calculations and flexible data caching approach used in pHusion.

For each region identified by the DoG, we cropped a small image series centered on the object in the raw image and extracted a defined number of frames before and after the event was detected. The size of the cropped image was 25×25 pixels for all of the data presented here, but is an adjustable parameter in pHusion. After applying a Gaussian filter of 1 pixel, a local background subtraction was performed using the median of the time points before detection and a Gaussian model was fitted to each cropped image (Fig. 1E). sepHluorin permits visualization of exocytic events due to the change in pH of the acidic vesicle lumen upon exposure to the extracellular environment; however, vesicles that are not sufficiently acidified will maintain the fluorescence of a distinct profile and must be ruled out from bona fide exocytic events. Instead of relying on particle-tracking algorithms to identify exocytic events as we did in ADAE GUI, here we developed a simple set of rules to determine whether an identified fluorescent event was a vesicle undergoing exocytosis.

We defined exocytic events to be the transient appearance of Gaussian fluorescence above a threshold that exhibited minimal drift in *x-y* and underwent exponential decay. These rules eliminate moving vesicles involved in trafficking, long-lasting fluorescent puncta that do not undergo decay and diffuse fluorescent fluctuations. To accomplish this, each potential event was evaluated for the intensity above background, the drift and duration. Furthermore, a measure of intensity (either the maximum or mean) was fitted with a two-step function consisting of a plateau followed by an exponential decay (Fig. 1F). To determine the length of the plateau, we calculated the goodness of fit (R^2^) for the function starting with no plateau and increasing in length until end of the image series. The best fit was selected. We chose to make these parameters as explicit and accessible as possible, allowing them to be easily adapted to different cell types and/or imaging conditions. With flexibility in mind, the code we have written in C^++^ enables modification of all key input parameters in ImageTank directly from the level of Gaussian blurring in the DoG, to the stringency of the drift calculation and the goodness of fit of the exponential decay (Table 1). To facilitate optimization and quality control, we report metrics for every region analyzed and return flags to indicate the reason for exclusion such as low intensity, drift or short duration. In addition to these quality control metrics, we report event characteristics, such as duration, intensity, decay rate and half-life (Table 2). Finally, despite best efforts, automated analysis will inevitably include some failures and thus we have included a manual override to either include erroneously eliminated events or remove false calls. This manually curated list is then used for further analysis, such as spatiotemporal analysis or classification. In its simplest form pHusion is performed on one timelapse movie at a time, although the analysis is amenable to batch processing, which can reduce the number of user interventions and greatly accelerate analysis time.

**Table 1.**
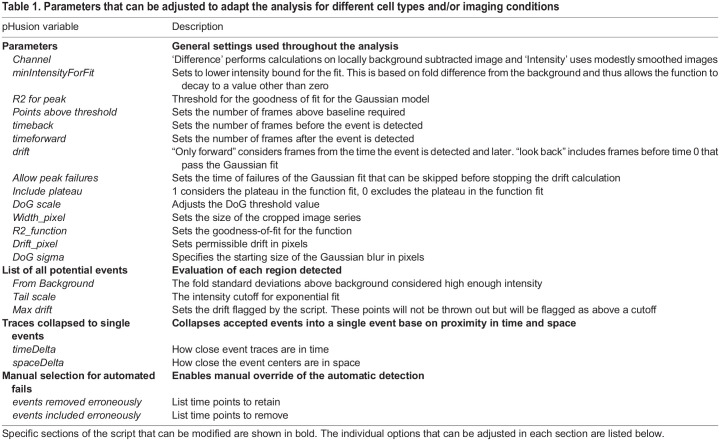
Parameters that can be adjusted to adapt the analysis for different cell types and/or imaging conditions

**Table 2.**
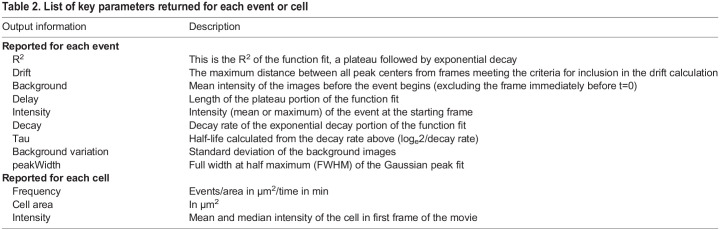
List of key parameters returned for each event or cell

### Comparison of ADAE GUI and pHusion

**Fig. 2.**
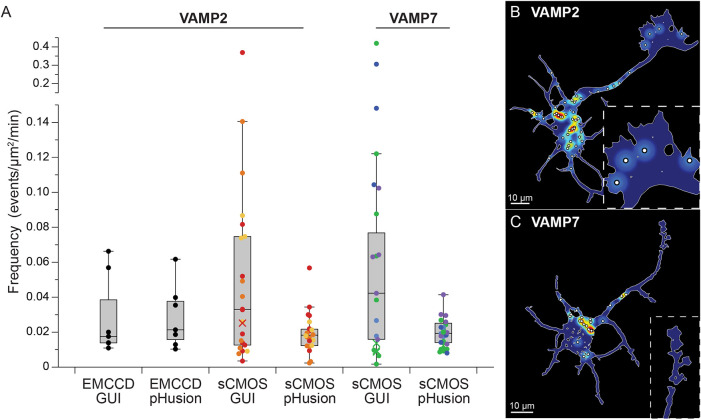
**Comparison of ADAE GUI and pHusion analysis.** (A) Primary murine cortical neurons at 2 days *in vitro* (DIV) expressing VAMP2–sepHluorin were imaged with either an EMCCD (*n*=7 cells) or sCMOS (*n*=24 cells) camera and analyzed with both the ADAE GUI and pHusion to automatically detect exocytic events. The frequency of exocytosis reported as mean±s.e.m. number of events/µm^2^/min were: EMCCD-GUI: 0.029±0.0087, EMCCD-pHusion: 0.029±0.0070, sCMOS-GUI: 0.058±0.017, sCMOS-pHusion: 0.024±0.002. VAMP7–sepHluorin-expressing cells (*n*=27) were imaged with a sCMOS camera and analyzed with pHusion (0.019±0.0015) and the GUI (0.079±0.021). On the figure, the box represents the 25–75th percentiles, and the median is indicated. The whiskers show the smallest and largest non-outlier values (within 1.5×IQR from the Q1 and Q3 boundaries). (B,C) Primary murine cortical neurons at 2 days *in vitro* (DIV) expressing VAMP2–sepHluorin (B) or VAMP7–sepHluorin (C) were imaged with sCMOS.

We were motivated to develop this more robust analysis tool because we found that ADAE GUI failed frequently for new users, in different cell types and/or for datasets acquired by distinct imaging paradigms. For example, when we changed our camera from an EMCCD to a high-speed sCMOS, the analysis failed completely for all movies over 4.29GB, as well as for unknown reasons on smaller datasets. For those cells for which results were returned, the set of frequencies calculated was far more varied with several clearly spurious results (Fig. 2A, sCMOS GUI). In contrast, when using pHusion, we found comparable exocytic frequencies in murine cortical neurons expressing VAMP2–sepHluorin imaged with EMCCD and sCMOS cameras that resulted in no failures regardless of camera type (Fig. 2A, pHusion).

VAMP7 is another vSNARE enriched in developing neurons that has been implicated in exocytosis and neuronal morphogenesis (Coco et al., 1999; Gupton and Gertler, 2010; Martinez-Arca et al., 2001; Urbina and Gupton, 2020). Our previous work has shown discrepancies in the frequency of exocytosis mediated by VAMP7 in developing cortical neurons with rates similar to VAMP2 reported for manually identified events (Gupton and Gertler, 2010; Winkle et al., 2014) and considerably lower frequencies detected with ADAE GUI (Urbina et al., 2018). We sought to analyze VAMP7–sepHluorin imaging with pHusion. Compared to VAMP2–sepHluorin, VAMP7–sepHluorin-expressing cells had a higher number of non-transient puncta and moving structures that confounded our analysis. This increase in fluorescent noise required us to adjust several parameters in the script to eliminate a great number of incorrectly identified events. To reduce the number of moving vesicles identified by pHusion, we tightened the permissible drift, included frames before the official start of the event provided they could be fitted with a Gaussian model and lowered the threshold for the goodness of fit for the model. Overall, these changes gave us more frames to track and improved our ability to remove these events. We also found that lowering the initial sigma value in the DoG reduced the number of diffuse puncta erroneously detected by the analysis. With these changes we reduced the error rate to under 10% and found the frequency of exocytosis to be similar to that of VAMP2–sepHluorin (Fig. 2A, VAMP7). Furthermore, consistent with our VAMP2–sepHluorin imaging, we found that analysis of VAMP7–sepHluorin-expressing cells with ADAE GUI failed to produce results for a number of cells and, when results were obtained, the frequencies were much more varied and in some cases clearly inaccurate (Fig. 2A, VAMP7 GUI). This provides a direct example of how the ability to adjust script parameters during data visualization benefits accurate analysis. We observed similar overall frequencies of exocytosis between VAMP2 and VAMP7. Mapping sites of exocytosis onto the neuronal mask also revealed more events occurring in the soma than the growth cone.

### Comparison of sepHluorin and pHmScarlet

**Fig. 3.**
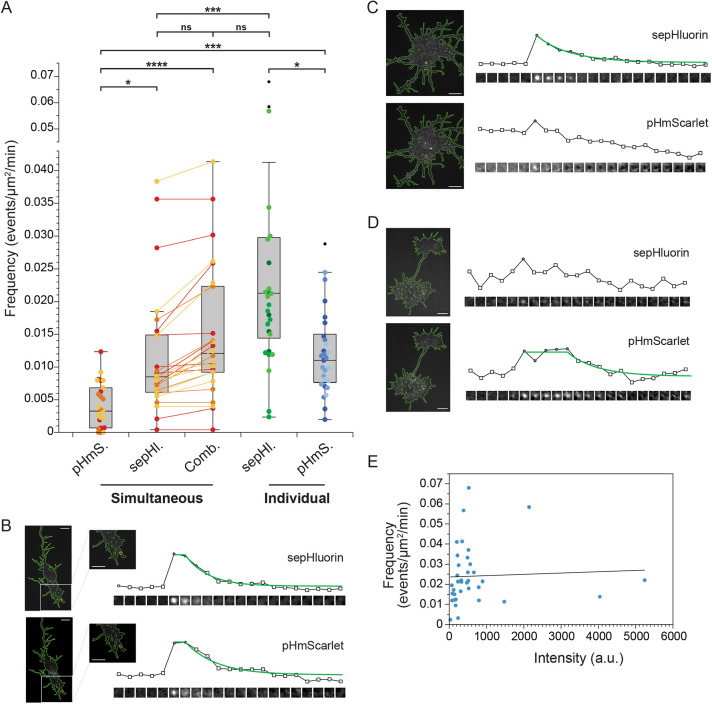
**Comparison of VAMP2–sepHluorin and VAMP2–pHmScarlet in developing cortical neurons.** (A) Frequency of exocytosis in murine neurons expressing both VAMP2–sepHluorin and –pHmScarlet (*n*=26) imaged simultaneously using a Hamamatsu Gemini Image Splitter and cells expressing VAMP2–sepHluorin alone (*n*=36) or VAMP2–pHmScarlet alone (*n*=27) imaged separately. In simultaneous imaging the frequencies, reported as mean±s.e.m. number of events/µm^2^/min, were pHmScarlet: 0.0041±0.00068, sepHluorin: 0.012±0.0019, or combined frequency of unique events in either channel: 0.015±0.0020. The frequency of VAMP2–sepHluorin imaged separately was 0.024±0.0024 and VAMP2-pHmScarlet was 0.012±0.0012.). On the figure, the box represents the 25–75th percentiles, and the median is indicated. The whiskers show the smallest and largest non-outlier values (within 1.5×IQR from the Q1 and Q3 boundaries). *P<0.05; ***P<0.001; ****P<0.0001; ns, not significant (nonparametric one-way ANOVA test). (B–D) Example image series centered on an event. The mean intensity in each image and the fit to the intensity values (green line) from the start of the event are plotted above. Circles indicate that the frame meets the criterium for a Gaussian function fit. A red circle marks the beginning of the event. Squares do not meet the criterium for a Gaussian function. (B) Representative event observed with both sepHluorin and pHmScarlet. (C) Representative event observed with sepHluorin but not pHmScarlet. (D) Representative event observed with pHmScarlet but not sepHluorin. (E) Plot of frequency (number of events/µm^2^/min) against intensity (a.u.) for cortical neurons expressing VAMP2-sepHluorin alone. The linear function fit is y=0.0235937+6.48894e-7*x with an R^2^ of 0.002. a.u., arbitrary units. All scale bars: 10 μm.

Our initial studies of exocytosis were done with sepHluorin, a well-established and widely used fluorophore in the green spectral range. We wanted to test a newly developed red pH-sensitive fluorophore called pHmScarlet (Liu et al., 2021) as it has been reported to be brighter than existing pH-sensitive red proteins like pHuji (Shen et al., 2014) and can capture both docking and fusion events (Liu et al., 2021). We imaged primary cortical neurons at DIV2 expressing either VAMP2–sepHluorin or VAMP2–pHmScarlet and found a significant, ∼50% reduction in detected exocytic frequency for pHmScarlet (Fig. 3A, Individual). For a more direct comparison, we imaged both probes simultaneously using a Hamamatsu Gemini Image Splitter and saw an even greater decrease in detected frequency (Fig. 3A, Simultaneous). This might be due in part to bleed-through correction required for the pHmScarlet channel when performing dual-color imaging. We found that ∼8% of the sepHluorin channel bled into the pHmScarlet image and therefore had to be corrected. However, we also detected significantly fewer events with each fluorophore expressed and imaged simultaneously when compared with the same reporter expressed and imaged individually (Fig. 3A). Interestingly, we observed that some events were captured with both sepHluorin and pHmScarlet (Fig. 3B), but a significant number of events were detected by only one reporter, either sepHluorin (Fig. 3C) or pHmScarlet (Fig. 3D). We quantified all unique events detected with either reporter and found that the combined frequency was not significantly different from that seen for VAMP2–sepHluorin imaged individually. Our data suggest that expressing both VAMP2 reporters simultaneously hampers the ability to detect either one as efficiently as imaging them independently. Furthermore, the frequency of exocytosis in cells expressing both VAMP2–sepHluorin and VAMP2–pHmScarlet is unlikely to be due to an overexpression artifact, as we found that the frequency of events does not depend on the expression level of VAMP2 (Fig. 3E).

### VAMP3-mediated fusion in oligodendrocytes and melanoma

**Fig. 4.**
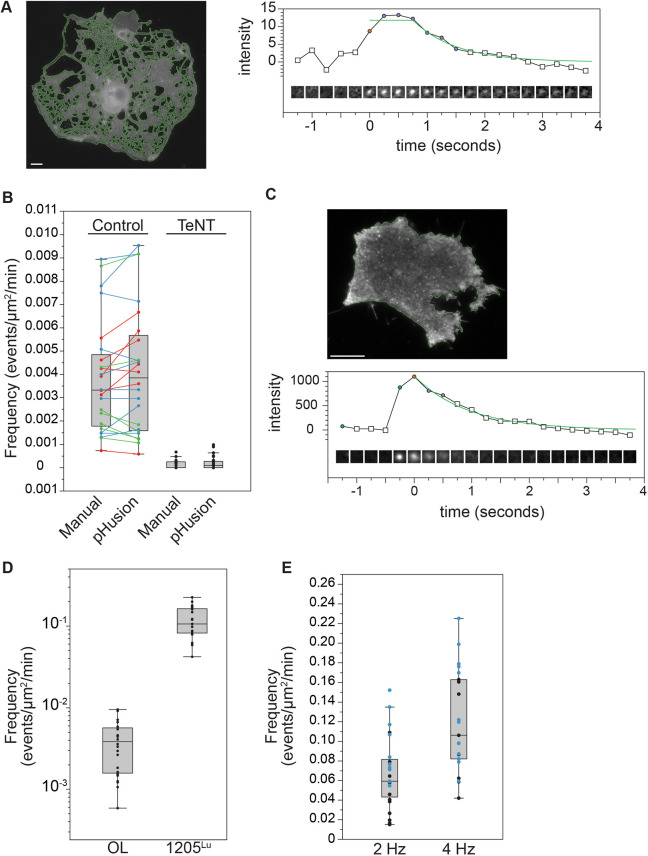
**The frequency of VAMP3 exocytosis in primary rat oligodendrocytes is much lower than that of human melanoma cells.** (A) Representative image of segmented oligodendrocyte (OL) and event detail. Scale bar: 10 µm. (B) Quantification of exocytosis with manual (0.0038±0.0005) and automated (0.0041±0.0006) detection (mean±s.e.m., *n*=21 cells). Frequencies reported as number of events/µm^2^/min. Cells expressing both VAMP3–sepHluorin and tetanus toxin light chain (TeNT) were quantified manually (0.00012±3.6×10^−5^) and automated (0.0002±5.6×10^−5^). (C) Representative image of 1205^Lu^ human melanoma cell line and example exocytic trace. Scale bar: 10 µm. (D) Frequency of VAMP3–sepHluorin exocytosis in primary rat OL (0.0041±0.0006) and 1205^Lu^ cells (0.12±0.011, mean±s.e.m., *n*=21). (E) Frequency of exocytosis in 1205^Lu^ cells imaged at 2 Hz (0.067±0.0077, mean±s.e.m., *n*=23) and 4 Hz (0.12±0.011, mean±s.e.m., *n*=21). For box plots, the box represents the 25–75th percentiles, and the median is indicated. The whiskers show the smallest and largest non-outlier values (within 1.5×IQR from the Q1 and Q3 boundaries).

To validate the versatility and flexibility of the exocytic detection further, we imaged primary rat oligodendrocytes at a mature stage of differentiation expressing VAMP3–sepHluorin using widefield epifluorescence microscopy. Mature oligodendrocytes are very large cells (∼120 µm in diameter) that require exocytosis during shape change (Lam et al., 2022) and are sufficiently flat to obviate the need for TIRF illumination. However, the background fluorescence was higher than that observed by TIRF imaging and the intensity of exocytic bursts was much lower (Fig. 4A). Surprisingly, despite these differences, we were able to adjust parameters in our script to successfully detect events. For oligodendrocytes, we increased the sigma for the DoG significantly and slightly lowered the stringency of the goodness of fit for the Gaussian model and the function fit. The frequency of exocytosis, although relatively low compared to cortical neurons at DIV2, was closely matched between automatic detection and manual analysis (Fig. 4B). To confirm that the events identified were bona fide exocytic events, VAMP3–sepHluorin was co-expressed with mRuby-tagged tetanus toxin light chain (TeNT), which cleaves VAMP3 and blocks VAMP3-mediated SNARE complex formation (Galli et al., 1994). Exocytosis was quantified by both manual and automated detection. Regardless of the analysis method, the frequency of exocytosis was reduced to negligible levels by co-expression of mRuby–TeNT (Fig. 4B), indicating identified events were bona fide exocytic events, and that the analysis pipeline in pHusion is sufficiently flexible for identification of exocytic events mediated by distinct vSNARES, in different cell types, imaged by widefield epifluorescence microscopy.

Secretion of matrix metalloproteases promotes metastasis of cancer cells. We next hoped to examine exocytosis in a metastatic cell line and determine how pHusion needed to be adapted for analysis of a mitotic, migratory cell type in which secretion is central to their pathology. We selected 1205^Lu^ cells, a melanoma cell line with a metastatic phenotype (Simon et al., 1996). Interestingly, in contrast to the modest modification our analysis script required to process oligodendrocytes imaged with epifluorescence, for images of VAMP3–sepHluorin in 1250^Lu^ cells acquired with the same TIRF imaging paradigm used above for cortical neurons, the script required significant alteration. A subset of small dim events of interest were lost during the DoG step due to high initial blurring, and thus we had to lower the sigma in the DoG to retain these events. Furthermore, the frequency of events was so high that the maximum intensity plot generated by the DoG did not have a clear demarcation between background and regions of interest. This required us to set a lower threshold for identifying regions for further evaluation in order to not miss events. However, lowering the threshold introduced fluorescent regions that were not exocytic events, and thus needed to be filtered. To do so, we tightened the stringency of the Gaussian fit and exponential decay to classify events as ‘true’. After making these adjustments, we achieved an error rate of less than 5% and found that the exocytic frequency of VAMP3 vesicles is far greater in human melanoma cells compared with primary rat oligodendrocytes (Fig. 4D). The frequency of exocytosis we report is likely an underestimation, as we found that increasing the speed of acquisition increased the frequency obtained, but we could not image faster than 4 Hz due to phototoxicity (Fig. 4E).

### Persistence of exocytic events in mature neurons

**Fig. 5.**
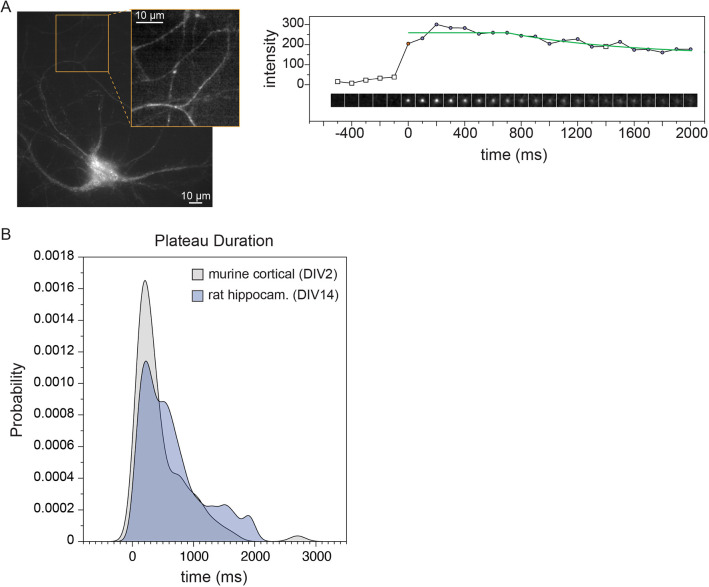
**Persistence of exocytic events in developing and mature neurons.** (A) Left, representative image of primary rat hippocampal neurons (DIV 12–14). The inset shows a detail of the network. Right, an example image series centered on an event. The mean intensity in each image and the fit to the intensity values (green line) from the start of the event are plotted above. Circles indicate that the frame meets the criterium for a Gaussian function fit. A red circle marks the beginning of the event. Squares do not meet the criterium for a Gaussian function. (B) Histograms of the time of the event plateau between mature (*n*=223) and immature neurons (*n*=203). Scale bars: 10 μm.

We also altered analysis parameters to enable automatic detection of exocytosis in mature primary rat hippocampal neurons (DIV12–14). Segmenting these highly complex and varied cells proved to be time consuming and error prone and thus we performed the analysis on the entire image without masking the cell (Fig. 5A). Interestingly, to image these fine, dim structures required camera settings that introduced shot noise that was detectable by the DoG analysis. These phenomena were exacerbated by the lack of a cell mask. Fortunately, shot noise is very rapid and does not move in *x-y* and thus could be eliminated based on duration and by requiring a minimum drift greater than zero. Furthermore, we observed that many events were persistent with a much longer plateau than those observed in developing cortical murine neurons (Fig. 5B). As a result, we had to lower the R^2^ threshold for the function fit. The R^2^ for a constant line is 0 and consequently inclusion of large segments of constant values artificially lowered the overall function fit. A list of key parameters altered to adapt pHusion to different datasets and the influence of these changes on the output is summarized in Table S1.

### Spatio-temporal analysis exocytosis

**Fig. 6.**
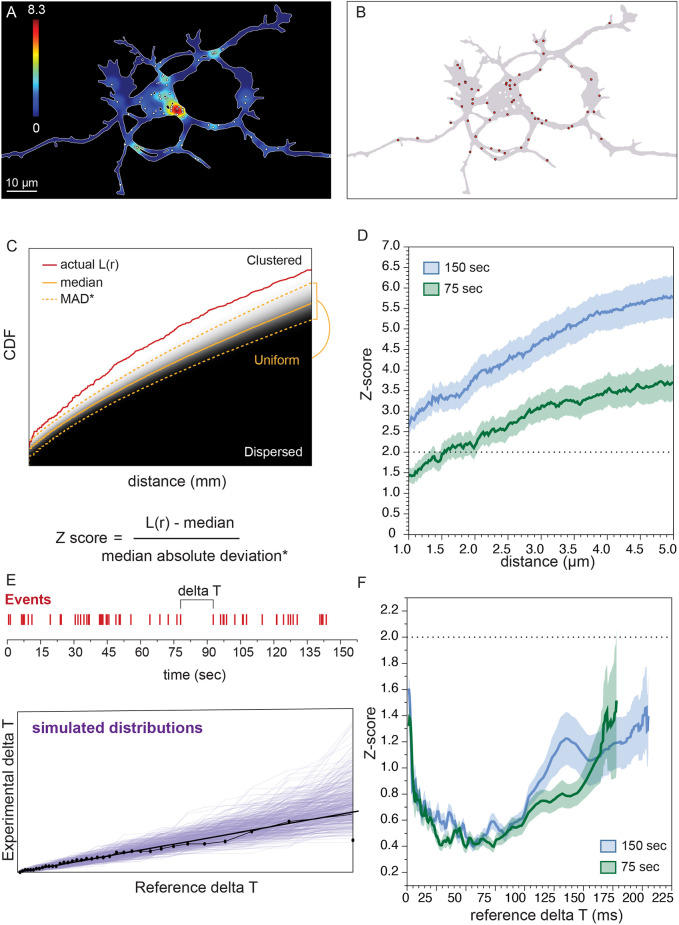
**Generalized analysis of spatial distribution of exocytosis.** (A) Representative heatmap of an actual event distribution. Scale bar: 10 μm. (B) Example of one Monte Carlo simulation of an event distribution using the same number of events detected and restricted to the cell mask. (C) The overlay of the cumulative density functions for all 25,000 simulations. The actual L(r) is shown in red, the median of the simulations is shown in orange and the bounds of the MAD are shown as orange dashed lines. An actual L(r) above the upper bound of the MAD is considered clustered, below the lower bound of the MAD is considered dispersed and between the bounds is taken to be uniform, or random. (D) Spatial Z-score (mean, with s.e.m. shown as shaded area) for VAMP2–sepHluorin exocytosis in developing murine cortical neurons for full length, 150 ms (*n*=45), or half length, 75 ms (*n*=36) movies. (E) Representative time series showing when events occurred and the time step (delta T) between events. The bottom panel shows a quantile-quantile (Q–Q) plot of the experimental delta T plotted against a standard exponential function with a decay rate of 1 (black dots). A line was fitted to this plot (black line). 200 simulated exponential distributions consistent with the fitted line are shown in purple. (F) Temporal Z-score (mean, with s.e.m. shown as shaded area) for VAMP2–sepHluorin exocytosis in developing murine cortical neurons for full length, 150 ms (*n*=45), or half length, 75 ms (*n*=36) movies.

Hotspots of vesicle fusion are frequently observed (Fig. 6A) in immature cortical neurons, suggesting that the spatial distribution of exocytic events was not random. Mathematically, random events are defined as being distributed uniformly, whereas dispersed events are distributed in a regularly spaced arrangement. Discerning whether exocytic events are clustered, uniform or dispersed requires spatial statistics. Our previous work to characterize the spatial distribution of exocytic events in developing neurons relied upon manual segmentation of the cell into the soma and neurites. We used Ripley's K analysis to classify events as clustered, dispersed or uniform (Urbina et al., 2018), and calculated an actual Ripley's L(r). Because this analysis was developed for simple geometries, we used a weighting term to compensate for edge effects in the neurites but not the soma.

Our goal in this work was to develop a generalized process to classify the spatial distribution of events that did not require manual input based on cell morphology. Using each cell mask and the number of events identified for that cell, we performed Monte Carlo simulations of events and plotted the cumulative distribution functions (CDF) for each of 25,000 simulations (Fig. 6B,C). Using this collection of CDFs, we calculated the median and the non-parametric median absolute deviation (MAD) as a function of distance (r) to define the boundaries, above which events are clustered and below which, events are dispersed (Fig. 6C). When distributed within the MAD, events are taken to be uniform or randomly distributed in space. The actual Ripley's L(r) for each cell was compared to its respective simulations. To enable comparison of a collection of cells regardless of differences in cell shape and the resulting CDFs, we calculated a non-parametric Z-score as a function of r by taking the difference between the actual L(r) and the median divided by the MAD. Z-scores above 2 (or below −2) are considered significant and are thus, not uniform. This criterium for the Z-score was chosen based upon the convention of two standard deviations away from the mean and is analogous to a *P*-value of 0.05. Although in our analysis we use the median and do not assume that data are normally distributed. Only cells in which 26 or more events were identified were included in our analysis due to noise in simulations of very sparse data. Consistent with previous work, we found that VAMP2-mediated events were clustered in space in developing cortical neurons at DIV2 (Fig. 6D). Interestingly, although we explicitly exclude time from the analysis, we do find that the duration of imaging does affect our results. For example, when we analyzed our full datasets acquired for ∼2.5 min, we observed spatial clusters over the entire range of distances considered. However, when we analyzed just the first half of the movies (∼1 min), we no longer observed spatial clustering of exocytosis over short distances (<1.5 µm), likely because we had not allowed sufficient time for repeated events to occur frequently enough in discrete hotspots.

In addition to measuring the spatial distribution of events, we wanted a robust method to determine whether exocytic events were random in time. Random events in time can be described as a Poisson process in which every event is independent of all others. By definition for a Poisson process measured over a given period of observation, the difference in time between all consecutive events will follow an exponential distribution. Thus, to determine whether exocytic events were random in time, we measured the time step (delta T) between consecutive pairs of events (Fig. 6E) and determined whether the set of delta T was exponentially distributed. To measure if a set of values was exponentially distributed, we used a quantile–quantile (Q-Q) plot of the experimental dataset against the standard exponential function with a decay rate of 1 (Fig. 6E**)**. When two datasets are drawn from the same underlying type of distribution, the Q-Q plot results in a line. Thus, we fitted a line to the Q-Q plot and simulated 200 exponential distributions consistent with the fit line. A simple linear regression was not robust enough to experimental noise to rigorously determine whether the data were consistent with an exponential distribution. Instead, the simulated distributions were used to generate an envelope against which experimental data could be tested. As with space, we calculated a Z-score across time by subtracting the fit line from the actual data and divided by the MAD. Z-scores above 2 were considered significant. To pool data from different cells each Z-score was interpolated onto a common time axis. We found that exocytosis occurred as random bursts in time regardless of the overall duration of the movie (Fig. 6F).

## DISCUSSION

Owing to the complexity and volume of image-based datasets generated by modern microscopy systems, computer-vision based approaches for analysis are warranted and, in some cases, essential. Although valuable, designing automated methods for quantification of visual data is very challenging and often results in brittle methods that work only for specific datasets. This is the problem we faced with automated analysis of exocytosis using ADAE GUI. Our initial processing scheme worked very well for the data on which it was developed, but failed frequently when used to analyze even slightly divergent datasets.

### Analysis improvements offered by pHusion

To develop a more versatile and robust application, we used ImageTank, software that seamlessly merges numerical methods and visualization of all data types from 3D image stacks to 1D data tables (Movie 1). The heavy reliance on visualization allowed us to rapidly detect key differences between datasets and identify ways to adapt the code for different datasets. Additionally, visualization provided built-in quality control mechanisms as the user can quickly see all aspects of workflow from the threshold applied to the DoG to each individual event evaluated. All of the C^++^ code is readily available and can be modified by the user to expand upon the metrics returned as needed. Visualization will also facilitate and guide the researcher in extracting additional information from their datasets. A key feature of our analysis pipeline is that data do not need to be passed between disparate applications, though both images and output tables can be exported in standard file formats such as .tiff and .xlsx. The ability to adapt analysis to different imaging conditions and consequently generate reliable annotated datasets can be useful in developing deep learning approaches.

Furthermore, improvements in camera technology both in terms of the speed of acquisition and the size of the chip has greatly enhanced resolution, but come at the cost of the ever increasing size of image files. To accommodate larger file sizes, we use a hybrid memory–disk based and ‘just-in-time’ computing approach that easily handles datasets too large to be stored in memory alone. With this data handling architecture, our script does not preclude analysis of large files well over the 4.29GB cutoff for so-called ‘big-tiff’ files. Moreover, where data are stored, on disk or in memory, is actively managed to increase efficiency and calculations are heavily threaded to greatly improve overall processing speed. One limitation to our reliance on ImageTank is that the graphics, user interface and threading rely heavily on the Mac operating system. Therefore, pHusion is unavailable for other operating systems such as Windows or Linux.

### Limitations and adjustments for analysis by pHusion

The method we have developed to identify exocytic events depends heavily on the DoG to highlight transient Gaussian fluorescence that rises above a baseline. In some datasets, such as developing neurons, the baseline of the maximum intensity plot of the DoG is apparent. However, this baseline can be obscured if events are too frequent or too rare. For example, we found that in 1205^Lu^ cells, events occurred so frequently that there were not enough timepoints without events, and thus the baseline could not be determined (Table S1). In this case, a much lower threshold had to be applied and care taken to filter out erroneously identified regions. Another difficulty with the DoG method can arise when very small regions of interest are analyzed with too infrequent numbers of events. For example, to visualize exocytosis in the growth cone, we performed the analysis on the whole cell and then segmented the growth cone instead of analyzing the growth cone by itself, as these data were too noisy and events too rare to accurately capture.

Our code will not be robust if events occur very frequently and very close together in space. We crop a small window around an event and assume that it is the only event in the window, that there is a peak associated with the event and that background intensity surrounds it. If two events are within the window they will likely be discarded. The size of the window can be adjusted, but if events occur extremely close in space and time, they might be difficult or impossible to resolve. Furthermore, some combinations of speeds that vesicles move at and the acquisition frame rate make exclusion of moving vesicles difficult. Adjusting the goodness of fit for the Gaussian function used to identify the center of the event, the number of frames required to track the center of an event and/or the permissible drift can improve detection. We recommend rigorously testing parameters on a subset of your data to find the best settings and then apply them to the full dataset.

In our hands, we find that the analysis in pHusion is versatile to a variety of expression levels of distinct fluorescent proteins, and that once an ideal parameter set is identified for a dataset, it will not need to be adjusted. Because the method subtracts a local background and looks at fold changes in intensity, not absolute values, detection is fairly robust. For example, we captured events in oligodendrocytes with intensities of ∼10 and in 1205^Lu^ of ∼1000 using the same criteria (fourfold intensity above background). If events in a new dataset are not sufficiently captured with the script, changing the fold intensity above background is an appropriate parameter to adjust, that is, lowering the criteria if events are much closer to the background, and increasing the criteria if there are bright transient fluctuations that are not exocytosis.

In addition to altering the parameters of our processing script to accommodate diverse datasets, adjusting the script for distinct imaging conditions was helpful and/or necessary as well. For example, although oligodendrocytes are sufficiently flat to enable epifluorescence imaging, the background was higher than in TIRF datasets. To address this, we photobleached VAMP3–sepHluorin within the PM prior to imaging exocytosis; we found this increase in the signal-to-noise ratio to be beneficial in imaging mature hippocampal neurons as well. Although useful, care must be taken to ensure cells are not damaged by phototoxicity as sensitivity to light varies greatly across cell types. For example, we were unable to image 1205^Lu^ melanoma cells as rapidly as neurons owing to the deleterious effects of light.

### Experimental adjustments for optimal analysis

Although pHusion is versatile in the variety of data and fusion events that it is capable of analyzing, some previous knowledge of the experiment model system is needed to obtain the best results. For example, the frequency and duration of the vesicle fusion events in the model system influence how rapidly images need to be acquired and for how long. Although the analysis script will run, the results will not be meaningful if images are not sufficiently fast to capture enough datapoints to fit a curve of a plateau and decay. The way functions are fitted will accommodate any physical timescale of data; however, the number of frames for an event is more important than the total time the event lasts in physical units. Some number of frames above background is required to distinguish events from noise (our analysis used three, but this is user-defined). However, if images are over sampled, this might reduce cell viability without increasing data quality. Also, if the plateau covers many frames, the length of the time series acquired might need to be increased and/or the parameters for the function fit adjusted, as long flat regions lower the R^2^ (Table S1).

Furthermore, how fast images need to be acquired relative to the frequency of events will depend on the type of information required. If frequency of events is the only desired output, then the Nyquist frequency (sampling at two times the frequency of events) is sufficient. If in contrast accurate information about the decay rate or plateau duration of the events is desired, then acquiring frames at a higher frequency than Nyquist is likely to be needed to have sufficient datapoints for a good fit. If sufficiently rapid imaging speed cannot be achieved due to technical limitations of the imaging system and/or cell health, adjusting the stringency of the R^2^ of the function fit can compensate up to a point. As indicated in Table S1, the R^2^ values used in this study spanned 0.24–0.75, depending upon the dataset.

### Using pHusion to assess new fluorescent proteins and exocytic clustering

We were interested in exploring a newly reported pH-sensitive fluorophore in the red spectral range, pHmScarlet, as this tool might enable us to distinguish docking and fusion events in exocytosis. Furthermore, the flexibility of multicolor imaging experiments is greatly enhanced by the availability of tools of different wavelengths. We found that we detected approximately half the number of events using VAMP2–pHmScarlet compared with when using VAMP2–sepHluorin when these probes were imaged separately, and an even greater decrease was seen when imaged simultaneously. However, due to bleed-through from sepHluorin into pHmScarlet, we removed 8% of the sepHluorin image from pHmScarlet, which lowered the overall intensity in that channel. Nevertheless, taken together our data suggest that pHmScarlet might underestimate the frequency of exocytosis, at least with our imaging setup, and that it only captures a subset of events.

Here, we presented a generalized approach to assess the spatial distribution of exocytic events that made no assumptions regarding cell shape. Additionally, we presented a robust method to determine whether events were random in time. These are useful tools in dissecting when and where exocytosis occurs and how it is regulated, but they are only a first step. In immature cortical neurons, we found events to be random in time. For circumstances in which events are not random in time, further work is required to dissect whether these events exhibit periodicity or non-random bursting. Furthermore, we have treated space and time independently. A more sophisticated approach would integrate space and time for a more complete picture of how exocytosis is regulated, but is beyond the scope of the current work.

## MATERIALS AND METHODS

### Software requirements and availability

ImageTank as a beta software is available to download from https://www.visualdatatools.com/ImageTank/. For the graphing functions in ImageTank a DataGraph license is needed. DataGraph is available with a trial and subscription options at https://www.visualdatatools.com/DataGraph/. Furthermore, to run pHusion GitHub Desktop (https://desktop.github.com) is required, as well as access to the public folder https://github.com/EllenClelia/exocytosis-to-share-with-DA and installation of Xcode (https://developer.apple.com/xcode/).

The pHusion script for single cells or batch processing and video tutorials can be found on the Gupton Lab website (https://guptonlab.web.unc.edu/phusion/).

### ImageTank analysis script

#### Difference of Gaussian

For the difference of Gaussian (DoG), images were blurred with increasingly large Gaussian filters starting with a sigma of 3 for cortical neurons expressing VAMP2–sepHluorin, 1 for cortical neurons expressing VAMP7–sepHluorin, 1 for 1205^Lu^ melanoma cells, 8 for oligodendrocytes and 3 for hippocampal neurons. An image stack based on 13 rounds (k) of blurring was generated by subtracting image k+1 from image k, and the median value of the image stack was calculated. The maximum intensity value in the median image was found for each time point. The median value of the maximum intensity plot was used to set the threshold above which identified regions of interest (ROI) for further evaluation. The threshold was adjusted by multiplying the median value by a scale factor. For cortical neurons and oligodendrocytes, the value was scaled by 1.2, in hippocampal neurons by 1.5 and in 1205^Lu^ cells by 0.7.

#### Evaluation of potential events

Using the ROI from the DoG, a small image series was cropped from the raw image stack taking a specified number of time frames before and after the spot was detected (defined as *t*=0). These smaller images were smoothed with a Gaussian filter of 1 pixel and were either used directly for subsequent analysis (channel ‘intensity’ in the parameters list) or were background subtracted (channel ‘difference’ in the parameters list). For background subtraction, the median of the images preceding *t*=0 was subtracted from the entire image series. The image just before the start of the event (*t*=−1) was excluded from the median as this often has elevated fluorescence intensity. Events occurring at the beginning the movie require at least two time frames to establish the background. Each image in the series was fitted with a Gaussian model. The R^2^ (goodness-of-fit) for the Gaussian model was set to 0.6 for cortical neurons, 0.5 for oligodendrocytes, 0.75 for 1205^Lu^ cells and 0.3 for hippocampal neurons. To be a valid event, the image at the timepoint the event was detected at must be above the R^2^ for the Gaussian model and only frames above this threshold were considered in the drift calculation. The centers of the Gaussian models were used to calculate the maximum distance between all valid frames. A measure of the intensity (maximum or average) was fitted with a two-step function consisting of a constant plateau and an exponential decay. The function need not decay to 0, the baseline is restricted by the parameter minIntensityForFit (Table 1). Events at the end of the movie can be captured provided there are sufficient frames to fulfill the input criteria including the number of frames above the background and the goodness of fit for the function. The R^2^ for the function fit was 0.24 for hippocampal neurons, 0.7 for cortical neurons, 0.75 for 1205^Lu^ cells and 0.5 for oligodendrocytes. The half-life, or tau, is calculated from the fit decay rate as ln(2)/decay rate, although this half-life only references the exponential decay portion of the curve.

#### Spatial analysis

Monte Carlo simulations using the number of events detected for a given cell and restricted to the mask were performed 25,000 times. The collective cumulative distribution functions of these simulations were used to find the median probability of events as a function of radius (r) for the specific geometry of the cell. The actual L(r) determined from real events was compared to the median as follows:

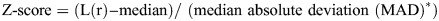


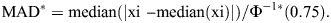


#### Temporal analysis

The time between sequential pairs of events were calculated for each cell to generate the delta T set. To assess whether the set of delta T values followed an exponential distribution used a Q-Q plot. For each cell, we sorted the delta T and assigned percentile values as follows:

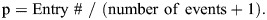
The actual delta T were plotted against theoretical delta T drawn from the standard exponential function with λ=1, f(t)=e^−t^. The theoretical delta T are calculated at the *P* values as follows:

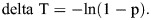
A variance weighted line was fitted to the resulting Q-Q plot and 400 exponential distributions were simulated that were consistent with the fit line to generate the Q-Q envelope. To compare the experimental data to the Q-Q envelope we used a Z-score similar to that calculated in space.

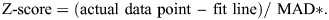
To collect all cells into an aggregate Z-score each individual Z-score was interpolated onto a standard time scale and the mean and s.e.m. were calculated for each time point.

Cells were excluded from both spatial and temporal analysis if fewer than 26 events occurred as the simulations were too noisy to be meaningful. However, pHusion will report results for cells with any given number of events (except 0) and thus the user can change the criteria for inclusion.

### Primary murine cortical neuron methods

#### Media, culture, transduction and transfection of primary murine cortical neurons

All mouse procedures were approved by University of North Carolina's Institutional Anima Care and Use Committee (IACUC) and followed the National Institutes of Health guidelines. C57Bl6 male and female embryos were used for primary cortical neuron preparations.

Cortical neuron cultures were prepared from day 15.5 embryos as previously described (Viesselmann et al., 2011). Briefly, cortices were dissected and neurons were dissociated with trypsin and plated on poly-D-lysine–coated glass-bottom culture dishes in neurobasal medium supplemented with B27 (Invitrogen). This same medium was used for all timelapse experiments. For all cells imaged with the cMos camera, VAMP2–SEpHluorin expression was mediated by adenoviral transduction, whereas for the EMCCD expression was mediated by nucleofection. Briefly, neurons were resuspended after dissociation in solution (Amaxa Nucleofector; Lonza) and electroporated with a nucleofector according to the manufacturer's protocol. For all other experiments exogenous expression was mediated by adenoviral transduction. Briefly, at 36–48 h before imaging 2–5 ml of concentrated virus was added to each dish of plated cells.

#### Adenovirus production

Adenovirus constructs were produced following the manufacturer's instructions [Takarabio Inc., Adeno-X™ Adenoviral System 3 (Tet-On® 3G Inducible)]. Briefly, Adeno-X 293 cells (Takarabio, cat. no. 632271) were transfected with linearized adenoviral vectors using Lipofectamine and Plus Reagent (Thermo Fisher Scientific). Cells were dislodged with gentle agitation upon exhibiting late cytopathic effect and were lysed with three freeze-thaw cycles. Pooled cell lysate and the flask supernatant was applied to a fresh flask of Adeno-X 293 cell for two rounds of amplification. Cell lysate alone was harvested by freeze-thaw cycles in PBS (Thermo Fisher Scientific, #MT-21-031-CV) following the final round of amplification. Expression of the target protein was induced with 1 µg/ml doxycycline (Sigma, D-9891).

#### Time-lapse imaging

All TIRF imaging was performed using an inverted microscope (IX83-ZDC2; Evident/Olympus) with Cellsens software (Evident/Olympus), a cMOS camera (Orca-Fusion, Hamamatsu) or an electron-multiplying charge-coupled device (iXon) where indicated, and a humidified live-cell imaging chamber (Tokai Hit Stage Top Incubator INUG2-FSX) maintained at 37°C and 5% CO_2_. DIV2 neurons expressing VAMP2–sepHluorin, VAMP2–pHmScarlet and/or VAMP7–sepHluorin in culture medium were imaged at 10 or 20 Hz with a 100×1.50 NA TIRF objective and a solid-state 491-nm laser illumination with 30% power and a 110-nm penetration depth and/or a solid-state 561-nm laser illumination at 30% power at 110-nm penetration depth. Simultaneous dual-color image acquisition was performed with the Hamamatsu W-VIEW GEMINI image splitter optic (A12801-01).

#### Statistical analysis

For two-sample comparisons of normally distributed data, an unpaired two-tailed *t*-test was used for two independent samples, or a paired two-tailed *t*-test for paired samples. For non-parametric data, we used the Kolmogorov–Smirnov (K-S) test to compare two-samples and the Kruskal–Wallis test for one-way ANOVA. Spatial and temporal analyses were tested with a non-parametric Z-score with a cut off of 2.

### Primary rat oligodendrocyte methods

#### Purification and culturing of primary oligodendrocytes

All rodent procedures were approved by Stanford University's Administrative Panel on Laboratory Animal Care (APLAC) and followed the National Institutes of Health guidelines. Sprague-Dawley rats were ordered from Charles River Laboratories. Both male and female rat pups were used for primary oligodendrocyte culture preparations.

Primary oligodendrocyte precursors were purified by immunopanning from post-natal day (P)5–P7 Sprague–Dawley rats as previously described (Dugas and Emery, 2013; Emery and Dugas, 2013). Oligodendrocyte precursors were initially seeded on 10-cm dishes coated with 0.01 mg/ml poly-D-lysine hydrobromide (PDL, Sigma P6407) at a density of 150,000–250,000 cells. Cells were allowed to recover for 4 days in DMEM-SATO proliferation medium [Dulbecco's modified Eagle's medium (DMEM); (Gibco/Life Technologies 11960044) containing 0.1 mg/ml BSA (Sigma-Aldrich A4161), 0.1 mg/ml transferrin (Sigma-Aldrich T1147), 16 μg/ml putrescine (Sigma-Aldrich P5780), 60 μg/mp progesterone (Sigma-Aldrich P8783), 40 μg/ml sodium selenite (Sigma-Aldrich S5261)], supplemented with 4.2 μg/ml forskolin (Sigma-Aldrich, cat. no. F6886), 10 ng/ml PDGF (Peprotech, cat. no. 100-13A), 10 ng/ml CNTF (Peprotech, cat. no. 450-02) and 1 ng/ml neurotrophin-3 (NT-3; Peprotech, cat. no. 450-03) at 37°C with 10% CO_2_ (Dugas and Emery, 2013; Emery and Dugas, 2013).

#### Transfection of oligodendrocyte precursors

Proliferating rat oligodendrocyte precursors were lifted from tissue culture dishes and centrifuged at 90 ***g*** for 10 min. 250,000 oligodendrocyte precursors were gently resuspended into 20 ml of nucleofector solution (Lonza P3 Primary Cell 4D-Nucleofector V4XP-3032) with 300 ng of VAMP3–sepHluorin and 300 ng of mRuby–caax or TeNT–mRuby–caax plasmids (see below). Cells were then electroporated in a Lonza 4D-Nucleofector X Unit (AAF-1003X) assembled with a 4D-Nucleofector Core Unit (AAF-1002B) with pulse code DC-218. Electroporated cells were rested for 10 min at room temperature (RT) before resuspension in antibiotic-free DMEM-SATO base medium supplemented with differentiation factors, containing 4.2 μg/ml forskolin (Sigma-Aldrich, cat. no. F6886), 10 ng/ml CNTF (Peprotech, Cat#450-02) and 40 ng/ml thyroid hormone (T3; Sigma-Aldrich, cat. no. T6397).

Each batch of 250,000 cells was distributed into four 35-mm dishes with no. 1.5 glass coverslips (MatTek, P35G-1.5-20-C) coated with PDL-borate (0.01 mg/ml PDL, which was first resuspended at 100× in 150 mM boric acid pH 8.4) for an optimal cell density. Each dish was half-fed with freshly supplemented DMEM-SATO every 2 days. After 5 days, medium was replaced with FluoroBrite DMEM-SATO (made with Thermo Fisher Scientific A1896701) supplemented with differentiation factors for 2 h at 37°C, 10% CO_2_ before imaging.

#### Live-cell imaging of exocytosis in primary oligodendrocyte-lineage cells

Time-lapse imaging of exocytic events was performed on a Zeiss Axio Observer Z1 inverted microscope equipped with a Zeiss Axiocam 506 monochrome 6-megapixel camera, a stage top incubator (Okolab, H301-K-Frame) set to 37°C and a digital gas blender (Okolab, CO2-UNIT-3L) set to 10% CO_2_ during image acquisition. Samples were imaged with a Plan-Apo 63×/1.40 oil objective using widefield epifluorescence with a 12 V halogen lamp. Due to a high baseline level of VAMP3–sepHluorin intensity on the cell surface, cells were subjected to initial ‘pre-bleaching’ consisting of 20 50-ms exposure at a 250-ms frame rate. Then, time-lapse sequences for exocytotic events were captured using an acquisition rate of 250 ms/frame for 1 min using the Zen Blue software. Images were viewed using Fiji/ImageJ software.

#### Cloning of plasmids used for oligodendrocyte transfections

For live imaging of exocytosis in cultured oligodendrocyte precursors and oligodendrocytes, the VAMP3–sepHluorin reporter consists of rat VAMP3 conjugated to sepHluorin cloned into a pAAV vector backbone with a CMV promoter (pCMV-VAMP3-pHluorin; Addgene #190152). DNA containing mRuby and tetanus toxin light chain (TeNT) were gifted from the Stanford Gene Vector and Virus Core and were cloned into a pAAV vector backbone with a myelin basic protein promoter (pMBP) (Gow et al., 1992). The pMBP drives expression of mRuby and/or TeNT at later stages (day 3+) of oligodendrocyte differentiation.

### Mature rat hippocampal neuron methods

#### Neuronal culture

Primary neuronal cells for culture were obtained in a similar procedure as previously described (Bingham et al., 2023). Briefly, hippocampi were extracted from E18 rat pups from pregnant female Wistar rats (Janvier labs), dissected, and homogenized by trypsin treatment followed by mechanical trituration. These were seeded on 18-mm diameter round, #1.5H coverslips at a density of 30,000 cells/cm^2^ for 3 h in serum-containing plating medium (MEM with 10% fetal bovine serum, 0.6% added glucose, 0.08 mg/ml sodium pyruvate, 100 UI/ml penicillin-streptomycin). In accordance with the Banker method (Kaech and Banker, 2006), the coverslips (cells facing down) were then transferred to and cultured in Petri dishes containing confluent glia cultures conditioned in NB+ medium (Neurobasal medium supplemented with 2% B-27, 100 UI/ml penicillin/ streptomycin and 2.5 µg/ml amphotericin).

All procedures were in agreement with the guidelines established by the European Animal Care and Use Committee (86/609/CEE) and was approved by the local French ethics committee (agreement G13O555).

#### Neuronal transfection and HILO microscopy

Neurons were transfected with 1 μg of VAMP2–sepHluorin between 12–14 DIV with Lipofectamine 2000 (Thermo Fisher Scientific). After 24 h, live-cell imaging of neurons expressing VAMP2–sepHluorin was performed on an inverted microscope ECLIPSE Ti2-E (Nikon Instruments) equipped with an ORCA-Fusion sCMOS camera (Hamamatsu Photonics K.K.; C14440-20UP) and a CFI SR HP Apochromat TIRF 100× oil (NA 1.49) objective. The system was equipped with a Nikon Perfect Focus System (PFS) and images were acquired with the NIS-Elements AR 5.30.05 software. Coverslips with neurons were mounted in a metal chamber in Tyrode's solution (in mM: 119 NaCl, 25 HEPES, 2.5 KCl, 2 CaCl_2_, 2 MgCl_2_, 30 glucose, pH 7.4). Neurons were maintained in a humid chamber at 35.5–37°C for the duration of the experiments using a stage-top incubator (Okolab inc). To image exocytosis, VAMP2–sepHluorin present initially in the plasma membrane was photobleached by exposing it to high power 488 nm laser light for 30 s before time-lapse imaging to remove the basal membrane signal and highlight exocytic signal (Yudowski et al., 2007); 100 ms exposure frames were then continuously acquired for 90 s using 488 nm laser light at lower power (1–10%).

#### Video preprocessing

Acquired videos were preprocessed using the Filter Timelapse script (available at https://sites.imagej.net/Christopheleterrier/plugins/NeuroCyto%20Lab/Kymographs/), which performed image stabilization using the Image Stabilizer plugin (https://imagej.net/plugins/image-stabilizer) after 2× downscaling, and bleach correction using average intensity compensation for the foreground identified as the top 12% of pixel intensities within each image of the sequence.

### Human melanoma cell line methods

#### Cell culture

1205^Lu^ cells were grown in DMEM (11995065; Gibco Thermo Fisher Scientific) supplemented with 10% FBS and 1% penicillin and 1% streptomycin, at 37°C, in a humified environment with 5% CO2.

#### Transfection and imaging

1205^Lu^ cells were plated on 35 mm glass bottom dishes with 20 mm micro-well #1.5 cover glass (D-35-20-1.5-n; Cellvis). For transient transfection, 1205^Lu^ cells were plated and 24 h later were transfected with a plasmid encoding VAMP3–sepHlourin using Lipofectamine (11668027; Life Technologies Inc) following the manufacturer's instructions. After 16–18 h, cells were imaged with a 100×1.49 NA TIRF objective and a solid-state 491-nm laser illumination at 35% power at 100-nm penetration depth. Images were acquired every 500 ms or 250 ms for 2 min with an exposure time of 50.995 ms. Cells showed signs of phototoxicity when imaged faster than 250 ms including cell rounding, developing vacuoles and loss of ruffling (Laissue et al., 2017).

## Supplementary Material



10.1242/joces.261828_sup1 Supplementary information
