# Supplementary material for: pHusion – a robust and versatile toolset for automated detection and analysis of exocytosis
Source: J Cell Sci. 2024 Jun 7;137(20):jcs261828. doi: 10.1242/jcs.261828 (PMC11190432; doi:10.1242/jcs.261828)
Supplement: Supplementary information [file joces-137-261828-s1.pdf]

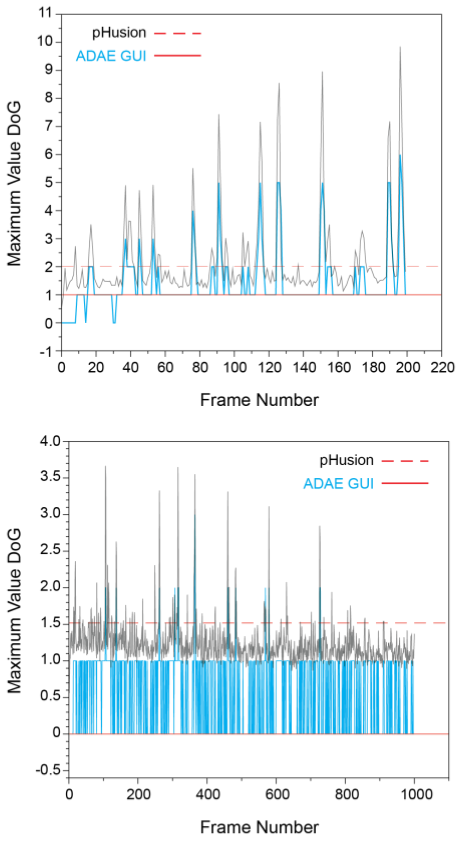

Fig. S1. Comparison of the Difference of Gaussian (DoG) maximum value plots calculated based on integers (ADAE GUI) or floating point numbers (pHusion) and the resulting thresholds applied (dashed and solid red lines). In the top panel, all of the major peaks are captured by both approaches and the resulting frequencies found are very similar: 0.062 (events/μm^2/min), pHusion, 0.066, ADAE GUI. In the bottom panel the threshold resulting from the ADAE GUI is 0 leading to a high number of false positives and markedly different frequencies: 0.058, pHusion, 0.37, ADAE GUI. Note, because the ADAE GUI converts images to 8-bit, to directly compare plots between platforms the images in pHusion were converted to 8-bit images. In our standard processing pipeline images are maintained as 16-bit and thus the resulting DoG plots are typically much greater than 1.

Journal of Cell Science • Supplementary information

Table S1. parameters set for different experimental samples and notes on how the input influences the analysis.

**Cell-type**		**Murine neurons DIV2**			**Rat neurons DIV 12-14**	**OL**	**Melanoma 1205^{Lu}**
**probe**		**VAMP2-pHluorin**	**VAMP2-pHmScarlet**	**VAMP7-pHluorin**	**VAMP2-pHluorin**	**VAMP3-pHluorin**	**VAMP3-pHluorin**
**Microscopy type**		**TIRF**	**TIRF**	**TIRF**	**HILO**	**Epi***	**TIRF**
**Pixel size (µm)**		0.065	0.065	0.065	0.13	0.072	0.065
**DoG sigma**	Sigma (in pixels) specifies the first level of Gaussian blur in a series of blurring and subtraction steps. Changing this value influences the size of features that are removed and emphasized (though intensity matters too). To remove small things increase the number and to preserve smaller objects lower it.	3	3	1	3	8	1
**DoG threshold**	This threshold determines which regions will be evaluated further. The number entered is a scale factor for the median value in the plot. Ideally a baseline will emerge above which transient bursts of intensity will be detected. This value should be set at the upper end of the baseline. If a clear baseline is not evident (melanoma cells) this value should be lowered to capture more ROIs and careful attention paid to what events are passing subsequent screening steps.	1.25	1.25	1.25	1.5	1.2	0.7
**Peak R^2**	Sets the goodness of fit for a Gaussian model of the peak. For a frame to be included in the drift calculation the peak as to pass this criteria. If there are a lot of moving objects in your movies you can lower this criteria to capture more frames for drift. You can also change the permissible drift (below) or consider frames before the official start of the event. Often the event is detected before the peak intensity is reached and these frames can be used to track movement as well.	0.7	0.6	0.6	0.3	0.5	0.75
**Function R^2**	Goodness of fit for the event function consisting of a plateau followed by exponential decay. If the plateau is long, your images are noisy or you have sparse coverage of the decay (not very many data points) this value can be lowered (as for rat neurons). Alternatively, if the DoG step leads to a high number of ROIs under investigation this value can be raised to tighten the criteria for	0.7	0.7	0.7	0.24	0.5	0.75

Journal of Cell Science • Supplementary information

	inclusion as a bona fide event (melanoma)						
**Drift_pixels**	Permissible drift. The script reports drift in physical units but it is often easier to see pixels and so we kept the option in this unit. This value can be changed depending on how many moving objects need to be removed, how stringent the criteria for the peak fit are, and how big the pixels are in physical units.	6	5	6	9*<br><br>*We required drift above 0 and cut off at 9	5	7
**Allow peak failures**	Allows a specified number of frames to fail the peak criteria when measuring drift. Often a single frame or two will be out of focus or disrupted in some way but overall the peak is still evident. This allows those failures to be disregarded and drift to be monitored over a longer time. If more than the specified number of frames fails the peak criteria the calculation stops at the last good frame.	1	1	2	0	0	1

*Cells were photobleached prior to imaging to enable epifluorescence imaging

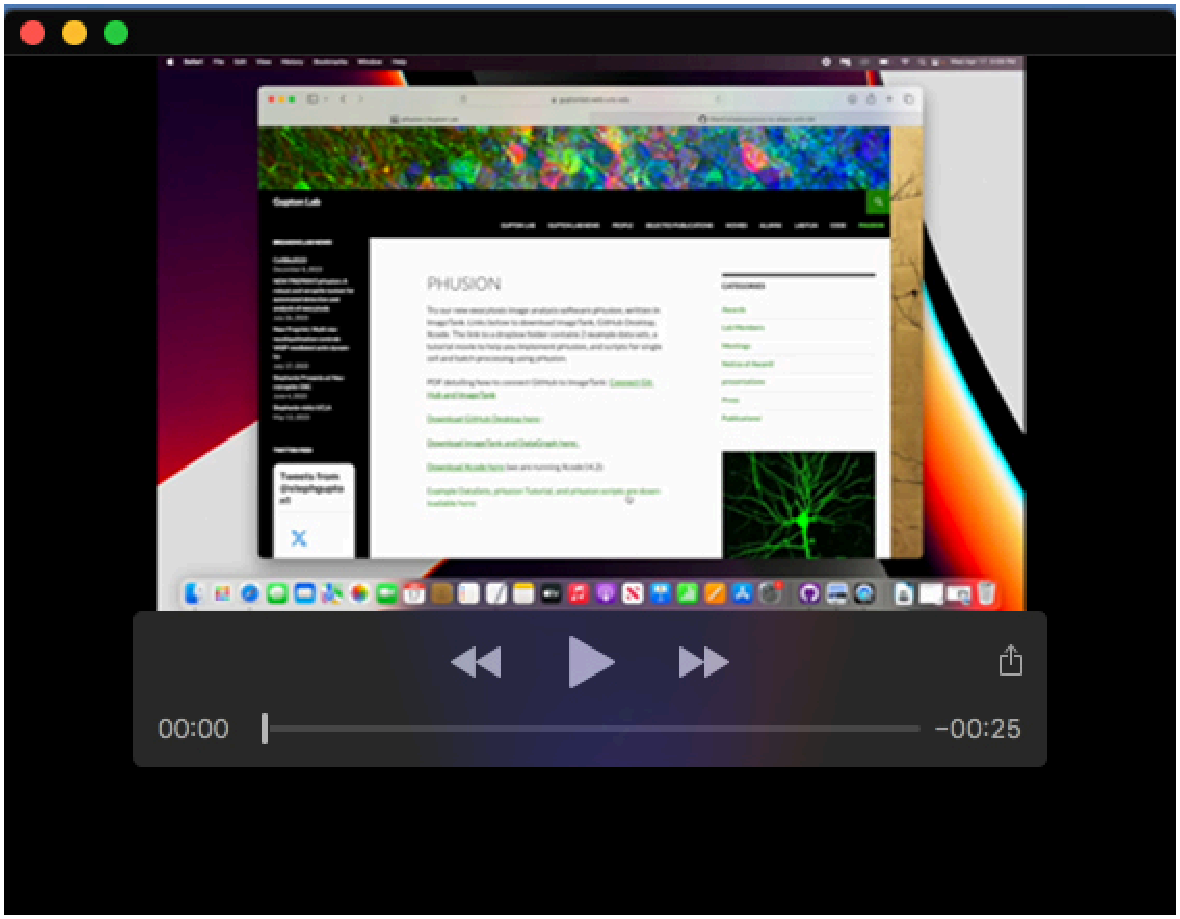

Movie 1. pHusion Tutorial. Explanatory tutorial for implementation of pHusion within ImageTank.

Journal of Cell Science • Supplementary information
