# Supplementary material for: pHusion – a robust and versatile toolset for automated detection and analysis of exocytosis
Source: J Cell Sci. 2024 Jun 7;137(20):jcs261828. doi: 10.1242/jcs.261828 (PMC11190432; doi:10.1242/jcs.261828)
Supplement: Supplementary file 1 [file joces-137-261828_review_history.pdf]

pHusion – a robust and versatile toolset for automated detection and analysis of exocytosis

Ellen C. O'Shaughnessy, Mable Lam, Samantha E. Ryken, Theresa Wiesner, Kimberly Lukasik, J. Bradley Zuchero, Christophe Leterrier, David Adalsteinsson and Stephanie L. Gupton
DOI: 10.1242/jcs.261828

Editor: Guillaume Jacquemet
Review timeline

Original submission:	23 November 2023
Editorial decision:	22 February 2024
First revision received:	18 March 2024
Editorial decision:	8 April 2024
Second revision received:	18 April 2024
Accepted:	24 April 2024

Original submission

First decision letter

MS ID#: JOCES/2023/261828

MS TITLE: pHusion: A robust and versatile toolset for automated detection and analysis of exocytosis

AUTHORS: Ellen O'Shaughnessy, Mable Lam, Samantha Ryken, Theresa Wiesner, Kimberly Lukasik, J. Bradley Zuchero, Christophe Leterrier, David Adalsteinsson, and Stephanie Gupton

ARTICLE TYPE: Tools and Resources

We have now reached a decision on the above manuscript.

To see the reviewers' reports and a copy of this decision letter, please go to: https://submit-jcs.biologists.org and click on the 'Manuscripts with Decisions' queue in the Author Area. (Corresponding author only has access to reviews.)

As you will see, the reviewers gave favourable reports but raised some critical points that will require amendments to your manuscript. I hope that you will be able to carry these out because I would like to be able to accept your paper, depending on further comments from reviewers.

Please ensure that you clearly highlight all changes made in the revised manuscript. Please avoid using 'Tracked changes' in Word files as these are lost in PDF conversion.

I should be grateful if you would also provide a point-by-point response detailing how you have dealt with the points raised by the reviewers in the 'Response to Reviewers' box. Please attend to all of the reviewers' comments. If you do not agree with any of their criticisms or suggestions please explain clearly why this is so.

Reviewer 1

© 2024. Published by The Company of Biologists under the terms of the Creative Commons Attribution License (https://creativecommons.org/licenses/by/4.0/).

Advance summary and potential significance to field

The manuscript by O'Shaughnessy and colleagues describes pHusion, an ImageTank-based pipeline for the analysis of exocytic events. From the results shown, pHusion does represent a significant improvement on their previous tool, ADAE GUI. PHusion built-in capabilities to tweak detection and tracking of events allows the analysis of exocytic events for a variety of cell types and exocytic markers imaged using different microscopy modalities.

Comments for the author

Major comments:
1) It is a real shame that the authors did not provide access to the software and a few sample movies for the reviewers. Personally, I would feel much more confident on praising the features of pHusion if I would have access to test it.
2) It is not clear what type of data pHusion outputs. The authors show in the manuscript mostly "frequency of events" and a one graph of plateau duration. Can users easily extract, for example, the half-life of recovery (tau of fusion events)? The manuscript (and the tool itself) would benefit if multiple types of outputs could be
extracted from the analyses.

Minor comments:
1) As movies were taken with slightly different frame rates, it is more meaningful to show time rather than frames for figures 4A, 4C and 5A. Also, consistency is advisable. The x-axis in Figure 1F is on ms while the axis for figures 4A, 4C and 5A are in relative frame number.
2) It would be good if the authors could make any conflicts of interest (CoI) clear on the manuscript. One of the authors is the owner of VDT, the company that developed ImageTank and I think there is a possible CoI (I am not a lawyer, I just want to be sure that all is done correctly) .

Reviewer 2

Advance summary and potential significance to field

The focus of this paper is the development of a single vesicle, membrane fusion analysis tool called "pHusion" for the automated identification of exocytosis events in live cells via fluorescence microscopy. Many labs use pH sensitive fluorescent probes and fluorescence microscopy to visualize fusion of vesicles with the plasma membrane. This fusion event coincides with a flash of fluorescence from pH dependent probes and this change in intensity is used to identify the time and location an exocytosis event occurred. This will be a very useful tool for the field and the authors have tested this on a variety of cell types and imaging conditions to verify the robustness of the analysis tool. The analysis tool is more robust and easier to adapt to different conditions because they use software that allows the person analyzing data to visualize the data easily (ImageTank). One downside of this software is the limitation to Mac computers but there are few (or possibly no) options that allow both high throughput analysis and visualization of the steps. If the tool is easy to adapt by other labs, this could be very helpful. Many of the minor comments below are meant to help other labs adapt the tool.

Comments for the author

Major Comments:
1) Although the authors are building off their past work and analysis of membrane fusion, they have missed analogous work done by others. This includes:
a. A synaptic activity automation (not listed in PubMed and difficult to find): Schmied C. et al SynActJ: easy-to-use automated analysis of synaptic activity. Front. Comput. Sci. 2021; 3
b. A semi-automated process in ImageJ for MVB fusion:
https://pubmed.ncbi.nlm.nih.gov/31836866/
c. A Matlab based automated process for MVB fusion:
https://pubmed.ncbi.nlm.nih.gov/36814381/
d. A new one in BioRxiv for insulin secretion from the Gandasi Lab. This does not need to be cited as it is not peer-reviewed, but I just wanted to share this with the authors.

© 2024. Published by The Company of Biologists under the terms of the Creative Commons Attribution License (https://creativecommons.org/licenses/by/4.0/).

https://doi.org/10.1101/2023.11.14.566999 e. The virology field has similar (in vitro) fusion assays but I am not sure if an analysis tool exists. This could mean that there are more people looking to use a tool like this.
2) The paper needs to be a bit more helpful for new users to adapt to using the analysis tool. The table is incredibly helpful in this respect as it lays out the parameters that are at play in the analysis. Could the authors state in the same table or an analogous one what they changed in the analysis for each cell type, camera, condition? This information is partly here in paragraphs or in the methods but placing it in one location would be easier for the reader.
For example, there are cells that have a higher background, and the analysis required a change in what parameter(s)? There are cells with “smaller” events and this required a lower DoG setting (maybe others).

Small things like this will help relieve the lab from having to assist and allow more uptake, but it is often difficult to see where this help is needed until others try to use it.

3) The paper was well-written and clear, with only a few spots where the authors possibly assume that the reader is more familiar with the past work than I am. I struggled a bit with the second to last paragraph of the results (Ripley’s K/L and MAD). Is there any reason for the setting -/+2 for the Z-score. What does a -2 mean? To use this tool, it seems that there’s a minimum amount of events per area required to determine hot spots. What is that? For example, one event in a cell will tell you nothing. Also note, that this section and the analysis of hotspots (temporal and spatial) is a specific strength to this tool and other analysis code listed above does not do this as well or at all.
4) It seems like the frequency of events, intensity of events, the duration of fusion events and the timing of the image acquisition would greatly affect the analysis.
a. What parameters depend on this? How would I adjust them? Again (like #2 above) I would place this into the table or a second table that summarizes how variables are changed for different parameters/cells/cameras etc.
b. What are the code’s limitations? What if an event is extremely slow? For example, different markers leave vesicles at different rates, as a user is there a right choice?
c. Do the decays need to reach 0 for analysis? If a fusion event happens at the end of movie, how is it counted? Do any of the parameters allow this to vary?
d. If I am transiently transfecting and see a variety of intensities across different cells because expression is heterogenous, will I need to change certain parameters each time? If so, which ones.

Minor comments:
5) The pHmScarlet is a factor of 2 less frequent than pHluorin. This is not small. You are missing half of the events with that probe (unless I am not understanding the data). I would state this amount in results and in the second to last paragraph of the discussion.
6) Include the pixel size (nm or um), sizes of vesicles that are fusing and how the DoG depends on this. For example, one cell type has smaller vesicles. As a reader, this confuses me because it comes after discussion of neurons which have very small, diffraction limited vesicles. Does this mean the camera and pixels are smaller or the actual vesicles are dimmer and appear smaller?
7) Does the analysis include the simulations described for the spatial or temporal hot spots or is this something that the authors have done alongside the pHusion tool?
8) Pre-processing – to be clear, this happens before using pHusion?
9) Intro – check the link to ImageTank and that it works. There’s a comma at the end.
10) What does a DoG of 1 mean? 1 pixel? pHusion workflow paragraph 2.
11) Same paragraph: does goodness of fit mean R^2 value?
12) Third paragraph of the pHusion workflow: “Using pHusion we identified consistent and comparable exocytic frequencies…” what is being compared here?
Different days, cells, etc?
13) In supplemental figure 1 and the discussion of the 8 bit data: it would be more appropriate to state or measure a signal to noise. It is not the fact that numbers are smaller that is the problem. It is that they are closer to the noise and S/N is smaller.
14) In the paragraph in results about pHmScarlet: data is either not different or different. If there is no significant difference then it isn’t “less than”.
15) Clarify in results what the “maximum intensity vector” is. I think the word vector is new here and it was a bit confusing. If it is what is plotted in Supplemental Figure 1, referring to that would help.

© 2024. Published by The Company of Biologists under the terms of the Creative Commons Attribution License (https://creativecommons.org/licenses/by/4.0/).

16) How do you measure phototoxicity? Please add one sentence to the methods.
17) Spatio-temporal analysis exocytosis section: "Hotspots of vesicle fusion are frequently observed…" are the authors referring to a certain cell type or all the cells or the ones discussed in the paragraph above. Please clarify.
18) Paragraph 4 of discussion: "Images need to have sufficient pixels to establish a baseline…" Could the authors be more specific? Even if the limits of this have not been tested, could you comment on what ranges have worked well for your analyses here?
19) End of discussion: underestimate is one word.
20) In a couple places the use of the word "brittle" to describe code confuses me. It seems redundant with the second phrase of both sentences. If brittle means something specific in coding, I could just be missing something.
21) One major strength of this analysis and the use of ImageTank is buried in the first paragraph of the results – there is no need to transfer data back and forth between programs. Consider mentioning this in the intro. This is a great feature!

First revision

Author response to reviewers' comments

We thank the reviewer for their thoughtful and helpful critiques of our manuscript and wonderful suggestions to make this more accessible to reviewers. We have addressed each point below and in the revised manuscript, and believe this makes the manuscript much stronger and pHusion much more available.

Reviewer 1 Advance Summary and Potential Significance to Field:
The manuscript by O'Shaughnessy and colleagues describes pHusion, an ImageTank-based pipeline for the analysis of exocytic events. From the results shown, pHusion does represent a significant improvement on their previous tool, ADAE GUI. PHusion built-in capabilities to tweak detection and tracking of events allows the analysis of exocytic events for a variety of cell types and exocytic markers imaged using different microscopy modalities.
Reviewer 1 Comments for the Author:
Major comments:
1)It is a real shame that the authors did not provide access to the software and a few sample movies for the reviewers. Personally, I would feel much more confident on praising the features of pHusion if I would have access to test it.

Thank you for this suggestion, it is a good point. We have created a registration link to ImageTank that is anonymous for reviewers. When prompted, enter this pass phrase "reviewerForGuptonPaper" in place of an email address and select the request button. Links to download ImageTank, DataGraph, GitHub, and Xcode can be found on the Gupton Lab website (https://guptonlab.web.unc.edu/phusion/). Further, pHusion scripts, instructions for how to set up the connect to GitHub desktop, how to load a new .tif movie and a more detailed tutorial video on pHusion are also available on our website. We also include a tutorial movie (Supplemental Video 1), to guide new users through implementation. At time of submission this was too large to upload to JCS, so please find link to this and example data also at the Gupton lab website listed above.

2)It is not clear what type of data pHusion outputs. The authors show in the manuscript mostly "frequency of events" and a one graph of plateau duration. Can users easily extract, for example, the half-life of recovery (tau of fusion events)? The manuscript (and the tool itself) would benefit if multiple types of outputs could be extracted from the analyses.

Thank you for this suggestion. The script outputs a number of parameters for each event analyzed (tau being one of them). We have now included a table summarizing the output of the code to the results section of the revised paper (**Table 2).** Further our intention is that individual users are able to edit the C++ code to alter the information returned. The source code for all steps performed with C++ is readily available on GitHub and accessible from within ImageTank/Xcode. Multiple avenues are available to export output from ImageTank. Images can be written as .tiff or .dtbin

© 2024. Published by The Company of Biologists under the terms of the Creative Commons Attribution License (https://creativecommons.org/licenses/by/4.0/).

(ImageTank file format) and tables can be exported as .xlsx or .dgraph. All data types can be passed to C++ or python.

The following text has been added to the discussion and instructions for how to extract data has been included in supplemental video 1:

“All of the C++ code is readily available and can be modified by the user to expand upon the metrics returned as needed…A key feature of our analysis pipeline is that data does not need to be passed between disparate applications though both images and output tables can be exported in standard file formats such as .tiff and .xlsx.”

Minor comments:
1)As movies were taken with slightly different frame rates, it is more meaningful to show time rather than frames for figures 4A, 4C and 5A. Also, consistency is advisable. The x-axis in Figure 1F is on ms while the axis for figures 4A, 4C and 5A are in relative frame number.

All figures have been converted to the physical time units.

2)It would be good if the authors could make any conflicts of interest (CoI) clear on the manuscript. One of the authors is the owner of VDT, the company that developed ImageTank and I think there is a possible CoI (I am not a lawyer, I just want to be sure that all is done correctly) .

This following text has been added to the manuscript after the acknowledgements:
“DA is the owner of Visual Data Tools Inc. developer of ImageTank and DataGraph.”

Below was added to the Materials & Methods:

“ImageTank as a beta software is available to download from https://www.visualdatatools.com/ImageTank/. For the graphing functions in ImageTank a DataGraph license is needed. DataGraph is available with a trial and subscription options at https://www.visualdatatools.com/DataGraph/. Further, to run pHusion GitHub Desktop (https://desktop.github.com) is required, access the public folder https://github.com/EllenClelia/exocytosis-to-share-with-DA and install Xcode (https://developer.apple.com/xcode/).”

*****

Reviewer 2 Advance Summary and Potential Significance to Field: The focus of this paper is the development of a single vesicle, membrane fusion analysis tool called “pHusion” for the automated identification of exocytosis events in live cells via fluorescence microscopy. Many labs use pH sensitive fluorescent probes and fluorescence microscopy to visualize fusion of vesicles with the plasma membrane. This fusion event coincides with a flash of fluorescence from pH dependent probes and this change in intensity is used to identify the time and location an exocytosis event occurred. This will be a very useful tool for the field and the authors have tested this on a variety of cell types and imaging conditions to verify the robustness of the analysis tool. The analysis tool is more robust and easier to adapt to different conditions because they use software that allows the person analyzing data to visualize the data easily (ImageTank). One downside of this software is the limitation to Mac computers but there are few (or possibly no) options that allow both high throughput analysis and visualization of the steps. If the tool is easy to adapt by other labs, this could be very helpful. Many of the minor comments below are meant to help other labs adapt the tool.

Reviewer 2 Comments for the Author:
Major Comments:
1)Although the authors are building off their past work and analysis of membrane fusion, they have missed analogous work done by others. This includes:
a.A synaptic activity automation (not listed in PubMed and difficult to find): Schmied C. et al SynActJ: easy-to-use automated analysis of synaptic activity. Front. Comput. Sci. 2021; 3
b.A semi-automated process in ImageJ for MVB fusion:
https://pubmed.ncbi.nlm.nih.gov/31836866/
c.A Matlab based automated process for MVB fusion: https://pubmed.ncbi.nlm.nih.gov/36814381/

© 2024. Published by The Company of Biologists under the terms of the Creative Commons Attribution License (https://creativecommons.org/licenses/by/4.0/).

d.A new one in BioRxiv for insulin secretion from the Gandasi Lab. This does not need to be cited as it is not peer-reviewed, but I just wanted to share this with the authors.
https://doi.org/10.1101/2023.11.14.566999
e.The virology field has similar (in vitro) fusion assays but I am not sure if an analysis tool exists. This could mean that there are more people looking to use a tool like this.

Thank you to reviewer two for pointing out these reference. We have included references to the first three peer reviewed, published articles with analysis tools in the revised manuscript.

The following text has been added to the Introduction:
"Given the fundamental importance of vesicle fusion and the availability of high quality image-based datasets, a number of tools have recently been published to analyze exocytosis in an automated (A, C) or semi-automated fashion (B). These powerful applications highlight the need for and interest in unbiased, computer-aided approaches to studying vesicle fusion that are also sufficiently robust for analysis of the diverse datasets. Our goal in the work presented here was to incorporate both the analysis we have come to rely on in our previous software to identify exocytic events with characterization of spatio-temporal dynamics in a single application capable of handling diverse datasets, obviating the need to pass data between software and improving visualization capabilities.

2)The paper needs to be a bit more helpful for new users to adapt to using the analysis tool. The table is incredibly helpful in this respect as it lays out the parameters that are at play in the analysis. Could the authors state in the same table or an analogous one what they changed in the analysis for each cell type, camera, condition? This information is partly here in paragraphs or in the methods but placing it in one location would be easier for the reader. For example, there are cells that have a higher background, and the analysis required a change in what parameter(s)? There are cells with "smaller" events and this required a lower DoG setting (maybe others).

Small things like this will help relieve the lab from having to assist and allow more uptake, but it is often difficult to see where this help is needed until others try to use it.
Yes! Thank you for this suggest, You are correct that we needed to do a better job of coalescing our experience with different datasets into a more user friendly format. In the revised manuscript we include a supplemental table (Supplemental Table 1) summarizing the key parameter options set for each experiment, descriptions of the dataset that may be helpful for new users to identify relevant changes for their own data, and notes on how this input influences the analysis. We also include a supplemental video 1 that will help new users implement pHusion.

3)The paper was well-written and clear, with only a few spots where the authors possibly assume that the reader is more familiar with the past work than I am. I struggled a bit with the second to last paragraph of the results (Ripley's K/L and MAD). Is there any reason for the setting -/+2 for the Z-score. What does a -2 mean? To use this tool, it seems that there's a minimum amount of events per area required to determine hot spots. What is that? For example, one event in a cell will tell you nothing. Also note, that this section and the analysis of hotspots (temporal and spatial) is a specific strength to this tool and other analysis code listed above does not do this as well or at all.

The bounds on the Z score +/- 2 is a standardized convention, analogous to a p-value of 0.05.

This text was added to the revised manuscript:
"This criteria for the Z score was chosen based upon the convention of two standard deviations away from the mean and is analogous to a p-value of 0.05. Though in our analysis we use the median and do not assume that data are normally distributed. Only cells in which 26 or more events were identified were included in our analysis due to noise in simulations of very sparse data."

Regarding the minimum number of events needed for determining clustering: This was instated due to noise - simulations of very sparse data are just not very meaningful. When we pooled our results, it was evident that very sparse cells were difficult to interpret and cells with at least 26 events were well behaved. 26 may seem odd but in time the analysis depends on a difference between events and thus 26 events gives 25 datapoints. However, the script always outputs results (unless there are truly 0 events) so the user can set a different cut off point or none at all.

© 2024. Published by The Company of Biologists under the terms of the Creative Commons Attribution License (https://creativecommons.org/licenses/by/4.0/).

The following text has been added to clarify this point in the results:
“Only cells in which 26 or more events were identified were included in our analysis due to noise in simulations of very sparse data.”

And this was added to the Materials and Methods:
“Cells were excluded from both spatial and temporal analysis if fewer than 26 events occurred as the simulations were too noisy to be meaningful. However, pHusion will report results for cells with any given number of events (except 0) and thus the user can change the criteria for inclusion.”

4)It seems like the frequency of events, intensity of events, the duration of fusion events and the timing of the image acquisition would greatly affect the analysis.

This is an interesting set of issues to ponder, and we have added a paragraph in the discussion that may assist the user in experimental design and analysis.

“Although pHusion is versatile in the variety of data and fusion events that it is capable of analyzing, some a priori knowledge of the experiment model system is needed to obtain the best results. For example the frequency and duration of the vesicle fusion events in the model system influence how rapidly images need to be acquired and for how long. Although the analysis script will run, the results will not be meaningful if images are not sufficiently fast to capture enough datapoints to fit a curve of a plateau and decay. The way functions are fit will accommodate any physical timescale of data, however the number of frames for an event is more important than the total time the event lasts in physical units. Some number of frames above background is required to distinguish from noise (our analysis used three, but this is user-defined). However, if images are over sampled, this may reduce cell viability without increasing data quality. Also, if the plateau covers many frames, the length of the time series acquired may need to be increased and/or the parameters for the function fit adjusted, as long flat regions lower the R^2 (Supplemental Table 1).”

a.What parameters depend on this? How would I adjust them? Again (like #2 above) I would place this into the table or a second table that summarizes how variables are changed for different parameters/cells/cameras etc.

We have added a supplemental table (Supplemental Table 1) listing the key parameters changed for different cell types/experiments and included a discussion of how these changes influence the analysis.

b.What are the code’s limitations? What if an event is extremely slow? For example, different markers leave vesicles at different rates, as a user is there a right choice?

These are also interesting points to consider. Some of these points are address in the paragraph above. In addition, we have included the following paragraphs to the Discussion to address limitations in the code and considerations regarding the frequency of events.

“Further, how fast images need to be acquired relative to the frequency of events will depend on the type of information required. If frequency of events is the only desired output, then the Nyquist frequency (sample at 2x the frequency of events) is sufficient. If in contrast accurate information about the decay rate or plateau duration of the events is desired, then frames acquired at a higher frequency than Nyquist is likely needed to have sufficient datapoints for a good fit. If sufficiently rapid imaging speed cannot be achieved due to technical limitations of the imaging system and/or cell health, adjusting the stringency of the R^2 of the function fit can compensate up to a point. As indicated in Supplemental Table 1 the R^2 values used in this study spanned 0.24-0.75, depending upon the dataset.“

“Our code will not be robust if events occur very frequently and very close together in space. We crop a small window around an event and assume that it is the only event in the window, that there is a peak associated with the event, and that background intensity surrounds it. If two

© 2024. Published by The Company of Biologists under the terms of the Creative Commons Attribution License (https://creativecommons.org/licenses/by/4.0/).

events are within the window they will likely be discarded. The size of the window can be adjusted, but if events occur extremely close in space and time, they may be difficult or impossible to resolve. Further, some combinations of speed that vesicles move and the acquisition frame rate make exclusion of moving vesicles difficult. Adjusting the goodness of fit for the Gaussian function used to identify the center of the event, the number of frames required to track the center of an event, and/or the permissible drift can improve detection. We recommend rigorously testing parameters on a subset of your data to find the best settings and then apply them to the full dataset."

c.Do the decays need to reach 0 for analysis? If a fusion event happens at the end of movie, how is it counted? Do any of the parameters allow this to vary?

The function does not need to decay to 0. The baseline is restricted by the parameter "minIntensityForFit" and is defined in Table 1. The following text was added to clarify that this value need not be "0"

minIntensityForFit	*Sets to lower intensity bound for the fit. This is based on fold difference from the background and thus allows the function to decay to a value other than zero*

And the following text was added to the Materials and Methods:
"The function need not decay to 0, the baseline is restricted by the parameter minIntensityForFit (Table 1)."

Regarding events that occur at the beginning or ending of the movie the following text has been added to the Material and Methods:
"Events occurring at the beginning the movie require at least two time frames to establish the background."

"Events at the end of the movie can be captured provided there are sufficient frames to fulfill the input criteria including the number of frames above the background and the goodness of fit for the function."

d.If I am transiently transfecting and see a variety of intensities across different cells because expression is heterogenous, will I need to change certain parameters each time? If so, which ones.

We find that the analysis is robust to differences in expression level. The following paragraph has been added to the discussion to clarify this point:

"In our hands, we find that the analysis in pHusion is versatile to a variety of expression levels of distinct fluorescent proteins, and that once an ideal parameter set is identified for a dataset, it will not need to be adjusted. Because the method subtracts a local background and looks at fold changes in intensity not absolute values detection is fairly robust. For example, we captured events in oligodendrocytes with intensities around 10 and in 1205[Lu] around 1000 using the same criteria (four fold intensity above background). If events in a new dataset are not sufficiently captured with the script, changing the fold intensity above background is an appropriate parameter to adjust, i.e. lowering the criteria if events are much closer to the background, and increasing the criteria if there are bright transient fluctuations that are not exocytosis."

Minor comments:
5)The pHmScarlet is a factor of 2 less frequent than pHluorin. This is not small. You are missing half of the events with that probe (unless I am not understanding the data). I would state this amount in results and in the second to last paragraph of the discussion.

You are correct, this text was added to both the results and discussion.

In the Results:
*"We imaged primary cortical neurons at DIV2 expressing either VAMP2-sepHluorin or VAMP2-pHmScarlet and found a significant, approximately 2-fold reduction in detected exocytic frequency for pHmScarlet (**Figure 3A, Individual**)."*

© 2024. Published by The Company of Biologists under the terms of the Creative Commons Attribution License (https://creativecommons.org/licenses/by/4.0/).

In the Discussion:
“We found that we detected approximately half the number of events using VAMP2-pHmScarlet compared with VAMP2-sepHluorin when these probes were imaged separately”

6)Include the pixel size (nm or um), sizes of vesicles that are fusing, and how the DoG depends on this. For example, one cell type has smaller vesicles. As a reader, this confuses me because it comes after discussion of neurons which have very small, diffraction limited vesicles. Does this mean the camera and pixels are smaller or the actual vesicles are dimmer and appear smaller?

The pixel sizes for each imaging setup has been added to Supplemental Table 1.

Thank you for pointing out this issue and forcing us to clarify our observations. In the melanoma cells a high number of small and dim events were lost due to how aggressively the images were blurred. They were lost because of a combination of their size and relative intensity above background. We altered the script to capture these events for subsequent evaluation. We do not have the resolution to determine the actual size of small the events, as you are correct, they are near the diffraction limit.

In addition to removing reference to events being smaller in diameter we added the following sentence to the Results section:
“A subset of small dim events of interest were lost during the DoG step due to high initial blurring, and thus we had to lower the sigma in the DoG to retain these events.”

7)Does the analysis include the simulations described for the spatial or temporal hot spots or is this something that the authors have done alongside the pHusion tool?

We have included the script with the revision and you can see that it is broken into two sections – the first for identifying and analyzing exocytosis and the second for spatio-temporal analysis. The simulations are done in pHusion. In space we use the number of events in the specific cell as well as the specific cell mask to restrict space for the simulations. We run a relatively large number of simulations to generate smooth CDFs to compare against but this is computationally very quick for such a simple simulation. In time we use the number of events to determine the how many points to include in an exponential curve and simulate 400 such curves to compare against.

The following text has been added to the Introduction to clarify that all analysis is done in a single application with no need to pass information between platforms:
“Our goal in the work presented here was to incorporate both the analysis we have come to rely on in our previous software to identify exocytic events with characterization of spatio-temporal dynamics in a single application capable of handling diverse datasets, obviating the need to pass data between software and improving visualization capabilities.”

8)Pre-processing – to be clear, this happens before using pHusion?

We are sorry for this confusion. All computational steps are performed in pHusion. You hand in only the raw images to the script.

We have added the following text to the Results section:
“We started with the basic framework for identifying potential exocytic events established in our previous work (Urbina et al., 2018) and perform all computational steps in pHusion”

9)Intro – check the link to ImageTank and that it works. There’s a comma at the end.

We tested it and it works on in our hands.

10)What does a DoG of 1 mean? 1 pixel? pHusion workflow paragraph 2.

We apologize for the confusion, sigma was not clearly defined in the text, the following sentence has been added to the Results:
“The initial level of blur (sigma) is specified by the user in pixels.”

© 2024. Published by The Company of Biologists under the terms of the Creative Commons Attribution License (https://creativecommons.org/licenses/by/4.0/).

11)Same paragraph: does goodness of fit mean R^2 value?

Yes. We are using the R^2 as the measure of goodness of fit (there are others but this is the most widely used). The following text has been added to the Results:

“we calculated the goodness of fit (R^2)”

12)Third paragraph of the pHusion workflow: “Using pHusion we identified consistent and comparable exocytic frequencies…” what is being compared here? Different days, cells, etc?

This text was added to clarify the comparisons we are making:

“In contrast, using pHusion we found comparable exocytic frequencies in murine cortical neurons expressing VAMP2-sepHluorin imaged with EMCCD and sCMOS cameras.”

13)In supplemental figure 1 and the discussion of the 8 bit data: it would be more appropriate to state or measure a signal to noise. It is not the fact that numbers are smaller that is the problem. It is that they are closer to the noise and S/N is smaller.

We apologize for the confusing manner this was originally described. The major difference between pHusion and the GUI is that the GUI uses integer values, whereas pHusion employs floating point numbers (decimals). When the calculations are performed with integers during preprocessing, median subtracting, and the DoG, many pixels become 0. A movie that produces too many 0 causes the DoG approach to fail. Because this does not happen with pHusion using decimal values, these failures do not occur.

For the purposes of comparing the two approaches, we wanted to overlay the maximum intensity plots of the DoG. Because part of the processing performed in the ADAE GUI converted all imaged to 8 bit, we converted images analyzed by pHusion to 8-bit as well.

The following text was clarified in the supplemental figure legend:
“Note, because the ADAE GUI converts images to 8-bit, to directly compare plots between platforms the images in pHusion were converted to 8-bit images. In our standard processing pipeline images are maintained as 16-bit and thus the resulting DoG plots are typically much greater than 1“

14)In the paragraph in results about pHmScarlet: data is either not different or different. If there is no significant difference then it isn’t “less than”.

Yes, you are right, the text now says:

“We quantified all unique events detected with either reporter and found that the combined frequency was not significantly different from VAMP2-pHluorin imaged individually.”

15)Clarify in results what the “maximum intensity vector” is. I think the word vector is new here and it was a bit confusing. If it is what is plotted in Supplemental Figure 1, referring to that would help.

We changed the word to plot in the Results:
“the maximum intensity plot generated by the DoG”

And describe in figure legend 1D:
“The maximum intensity in the DoG image plotted over time”

16)How do you measure phototoxicity? Please add one sentence to the methods.

This sentence was added to the M&M:
“Cells showed signs of phototoxicity when imaged faster than 250ms including cell rounding, developing vacuoles and loss of ruffling (Laissue et al., 2017)”

© 2024. Published by The Company of Biologists under the terms of the Creative Commons Attribution License (https://creativecommons.org/licenses/by/4.0/).

17)Spatio-temporal analysis exocytosis section: “Hotspots of vesicle fusion are frequently observed…” are the authors referring to a certain cell type or all the cells or the ones discussed in the paragraph above. Please clarify.

Yes, good point. This work was done in immature cortical neurons. The following text has been added to the Results:

“Hotspots of vesicle fusion are frequently observed (Figure 6A) ***in immature cortical neurons****, suggesting that the spatial distribution of exocytic events was not random.”*

18)Paragraph 4 of discussion: “Images need to have sufficient pixels to establish a baseline…” Could the authors be more specific? Even if the limits of this have not been tested, could you comment on what ranges have worked well for your analyses here?

This is a very difficult question to answer in a general way as it depends on many aspects that will be unique to each experimental setup – how frequent are the events, what is your frame rate, what is the background distribution of the probe and what is the noise inherent in the imaging setup? In looking at our data on the growth cones again we realize that the difficulty in establishing a baseline comes from both how few pixels are in the ROI and how infrequent the events are. Because both of these things will vary in different experiments we cannot define a “how small” criteria. To clarify these points and provide guidance on establishing a baseline the following text has been added to the Discussion”

“The method we have developed to identify exocytic events depends heavily on the DoG to highlight transient Gaussian fluorescence that rises above a baseline. In some datasets, such as developing neurons, the baseline of the maximum intensity plot of the DoG is apparent. However this baseline can be obscured if events are too frequent or too rare. For example, we found that in 1205^{Lu} cells, events occurred so frequently that there were not enough timepoints without events, and thus the baseline could not be determined (Supplemental Table 1). In this case a much lower threshold had to be applied and care taken to filter out erroneously identified regions. Another difficulty with the DoG method can arise when very small regions of interest are analyzed with too infrequent of events. For example, to visualize exocytosis in the growth cone we performed the analysis on the whole cell and then segmented the growth cone instead of analyzing the growth cone by itself, as these data were too noisy and events too rare to accurately capture.”

19)End of discussion: underestimate is one word.

This was corrected in the text.

20)In a couple places the use of the word “brittle” to describe code confuses me. It seems redundant with the second phrase of both sentences. If brittle means something specific in coding, I could just be missing something.

We were using the term “brittle” in regard to code. Brittle code breaks with small changes. The following text was added to the Introduction to clarify this point:

“brittle, failing frequently, often for very minor changes in experimental data”

21)One major strength of this analysis and the use of ImageTank is buried in the first paragraph of the results – there is no need to transfer data back and forth between programs. Consider mentioning this in the intro. This is a great feature!

Thank you for this suggestion! This sentence was added to the introduction:
“An advantage of this approach is that data is not transferred manually between separate applications as in our previous method thereby reducing the risk of error and simplifying the process for the user.”

© 2024. Published by The Company of Biologists under the terms of the Creative Commons Attribution License (https://creativecommons.org/licenses/by/4.0/).

Second decision letter

MS ID#: JOCES/2023/261828

MS TITLE: pHusion: A robust and versatile toolset for automated detection and analysis of exocytosis

AUTHORS: Ellen O'Shaughnessy, Mable Lam, Samantha Ryken, Theresa Wiesner, Kimberly Lukasik, J. Bradley Zuchero, Christophe Leterrier, David Adalsteinsson, and Stephanie Gupton

We have now reached a decision on the above manuscript.

To see the reviewers' reports and a copy of this decision letter, please go to: https://submit-jcs.biologists.org and click on the 'Manuscripts with Decisions' queue in the Author Area. (Corresponding author only has access to reviews.)

As you will see, the reviewers gave favourable reports but raised some minor points that will require amendments to your manuscript. I hope that you will be able to carry these out because I would like to be able to accept your paper.

Please ensure that you clearly highlight all changes made in the revised manuscript. Please avoid using 'Tracked changes' in Word files as these are lost in PDF conversion.

I should be grateful if you would also provide a point-by-point response detailing how you have dealt with the points raised by the reviewers in the 'Response to Reviewers' box. Please attend to all of the reviewers' comments. If you do not agree with any of their criticisms or suggestions please explain clearly why this is so.

Reviewer 1

Advance summary and potential significance to field

Phusion is a very useful tool for the cell biology community. The possibility to identify and quantify various types of exocytic events, from different cells and using different microscopy methods represents a real advance. It is just a real shame it is exclusive for Mac users, which excludes most labs in the global south and/or with limited funding.

Comments for the author

I thank the authors for addressing all my comments and providing access to the code and sample movies for testing. I am satisfied with the corrections and the script is indeed a great tool.

As ImageTank is a very niche software, It would be great if the authors would include in the first 1 or 2 minutes of the tutorial a few words on imageTank and the process of installation, registration, connection to GitHub and opening Phusion. Having this info spread on multiple places is not user friendly. Moreover, the tutorial should not start by saying "This is what fusion will look like AFTER you analysed the cell". The initial reaction for most users would be "how did you get there?". As this was my first contact with ImageTank, it took me at least 30 minutes to realise that I had to drag the .itank file into the software rather than building the analysis up from the "external task listing". This was the impression I got from the document explaining how to link imageTank to GitHub.

Reviewer 2

Advance summary and potential significance to field

The focus of this paper is the development of a single vesicle, membrane fusion analysis tool called “pHusion” for the automated identification of exocytosis events in live cells via fluorescence

© 2024. Published by The Company of Biologists under the terms of the Creative Commons Attribution License (https://creativecommons.org/licenses/by/4.0/).

microscopy. This will be a very useful tool for a wide range of fields and the authors have tested this under a variety of conditions. The authors changes will likely make the tool easier to adapt by other labs.

Comments for the author

No additional changes are necessary. All the past comments were appropriately addressed in the revision.

Second revision

Author response to reviewers' comments

We have adjusted the video with the requested changes. I didn't reupload the supplemental from before, that the mnauscript and figures are all the same. the only change is to the video

---

Third decision letter

MS ID#: JOCES/2023/261828

MS TITLE: pHusion: A robust and versatile toolset for automated detection and analysis of exocytosis

AUTHORS: Ellen O'Shaughnessy, Mable Lam, Samantha Ryken, Theresa Wiesner, Kimberly Lukasik, J. Bradley Zuchero, Christophe Leterrier, David Adalsteinsson, and Stephanie Gupton

I am happy to tell you that your manuscript has been accepted for publication in Journal of Cell Science, pending standard ethics checks.

© 2024. Published by The Company of Biologists under the terms of the Creative Commons Attribution License (https://creativecommons.org/licenses/by/4.0/).
